# Characterization of carp seminal plasma Wap65-2 and its participation in the testicular immune response and temperature acclimation

**DOI:** 10.1186/s13567-020-00858-x

**Published:** 2020-11-25

**Authors:** Mariola A. Dietrich, Mikołaj Adamek, Verena Jung-Schroers, Krzysztof Rakus, Magdalena Chadzińska, Anna Hejmej, Piotr Hliwa, Barbara Bilińska, Halina Karol, Andrzej Ciereszko

**Affiliations:** 1 Department of Gamete and Embryo Biology, Institute of Animal Reproduction and Food Research, Polish Academy of Sciences, Tuwima 10, 10-748 Olsztyn, Poland; 2 Fish Disease Research Unit, Institute for Parasitology, University of Veterinary Medicine Hannover, Buenteweg 17, 30559 Hannover, Germany; 3 Department of Evolutionary Immunology, Institute of Zoology and Biomedical Research, Faculty of Biology, Jagiellonian University, Gronostajowa 9, 30-387 Krakow, Poland; 4 Department of Endocrinology, Institute of Zoology and Biomedical Research, Faculty of Biology, Jagiellonian University, 30-387 Krakow, Poland; 5 Department of Ichthyology and Aquaculture, University of Warmia and Mazury in Olsztyn, Warszawska 117A, 10-701 Olsztyn, Poland

**Keywords:** acclimation, cDNA, fish, hemopexin, infection, reproductive system, semen, Wap65

## Abstract

Two functionally distinct isoforms of warm-temperature acclimation related 65-kDa protein (Wap65-1 and Wap65-2) with a role in the immune response are present in fish. To our knowledge, contrary to Wap65-1, Wap65-2 has neither been isolated nor functionally characterized in carp especially in reproductive system. The aim of this study was to characterize Wap65-2 and ascertain its functions in immune response and temperature acclimation within reproductive system. Wap65-2 corresponded to one of the most abundant proteins in carp seminal plasma, with a high immunologic similarity to their counterparts in seminal plasma of other fish species and a wide tissue distribution, with predominant expression in the liver. The immunohistochemical localization of Wap65-2 to spermatogonia, Leydig cells, and the epithelium of blood vessels within the testis suggests its role in iron metabolism during spermatogenesis and maintenance of blood-testis barrier integrity. Wap65-2 secretion by the epithelial cells of the spermatic duct and its presence around spermatozoa suggests its involvement in the protection of spermatozoa against damage caused by heme released from erythrocytes following hemorrhage and inflammation. Our results revealed an isoform-specific response of Wap65 to temperature acclimation and *Aeromonas salmonicida* infection which alters blood-testis barrier integrity. Wap65-2 seems to be related to the immune response against bacteria, while Wap65-1 seems to be involved in temperature acclimation. This study expands the understanding of the mechanism of carp testicular immunity against bacterial challenge and temperature changes, in which Wap65-2 seems to be involved and highlights their potential usefulness as biomarkers of inflammation and temperature acclimation.

## Introduction

Fish warm-temperature acclimation related 65 kDa protein (Wap65) is an ortholog of hemopexin (Hpx, alternative name beta-1B-glycoprotein), a protein that has been identified in all vertebrates [1]. Hpx is a plasma glycoprotein that binds to heme with high affinity and transports heme to hepatocytes, where it is internalized and degraded [2]. Hpx is recognized as the major heme-scavenging protein in the plasma; it prevents free heme-mediated cellular damage [1]. Heme intercalates into lipid membranes and participates in the formation of highly-toxic hydroxyl radicals and oxidation of low-density lipoprotein (LDL) cholesterol [3]. Hpx also acts as a multifunctional agent in iron homeostasis, bacteriostatic defense (limiting the access of pathogens to heme), antioxidant protection, nerve regeneration, and gene expression to promote cell survival [4].

In fish, Wap65 was initially identified in goldfish muscle and hepatopancreas after thermal acclimation [5–8] and has since been cloned from a large number of fish [9–25]. Notably, Wap65 function is mainly related to warm-temperature acclimation, which is most significant in eurythermal fish such as carp that live over a wide range of temperatures, from near zero to over 30 °C [5–7, 9, 10, 14, 15, 20, 22]. Besides, Wap65 is involved in the immune response [8, 14, 17, 18, 21–24, 26], intoxication [13, 19, 25], and embryo development [11, 12, 16].

In contrast to a single isoform of Hpx in mammals, there are two functional Wap65 isoforms in fish (Wap65-1 and Wap65-2) [10–12, 14, 16, 18, 19, 21–25]. These isoforms display distinct tissue distribution patterns and physiological functions in response to stimuli. In general, Wap65-1 is widely expressed and strongly regulated by an increase in temperature, while Wap65-2 has a restricted tissue distribution (mainly in the liver) and is more regulated by bacterial infection rather than warm-temperature stress; that fact indicates its role in immune response in several teleosts [18–20, 22–24]. Notably, the expression patterns of Wap65-1 and Wap65-2 and response to different stimulatory treatments are variable and contradictory depending on the fish species [10–12, 14, 21, 23, 25].

Kinoshita et al. [9] isolated Wap65-1 from carp serum and determined its full cDNA sequence. The main function of Wap65-1 has been attributed to a response to warm-temperature acclimation [9]. By using a proteomic approach, we recently identified both Wap65-1 and Wap65-2 in carp blood plasma and reported their involvement in temperature acclimation [27]. Moreover, we described the presence of Wap65-1 in carp seminal plasma [28]. However, we were unable to identify Wap65-2, likely due to the lack of its sequence in databases. To our knowledge, contrary to Wap65-1, Wap65-2 has neither been isolated nor characterized in carp. This step is prerequisite to obtain information concerning potential physiological functions of Wap65-2 in carp, especially to verify its function in the reproductive system and the immune response.

Although the testis is an immuneprivileged site where systemic immune responses are remarkably reduced, the testis has effective local innate immunity against invading pathogens derived from both the circulating blood and via the ascending male genitourinary tract. Fish have shown reproductive stage-dependent expression patterns of immune-relevant genes in testis; these data suggest that they function in the protection of developing germ cells against infection [29]. Moreover, the dominance of proteins (including Wap65) involved in the immune and stress response in fish seminal plasma [30] indicates their pivotal role in the protection of spermatozoa against infection and inflammation. However, their specific functions in testicular immunity have been not reported, except the bacteriostatic activity of Kazal type 2 and Apo-A1 [31, 32].

In the current study, we isolated and characterized Wap65-2 from carp seminal plasma and obtained its full-length cDNA sequence. Furthermore, we determined the tissue distribution profiles of Wap65-2 in relation to Wap65-1 using quantitative real-time polymerase chain reaction (qPCR). We employed immunohistochemistry to examine Wap65-2 protein expression within the reproductive system. In experiments directed toward better understanding biological functions in relation to temperature acclimation (10 °C and 30 °C) and immune response against bacteria (*Aeromonas salmonicida*), we compared mRNA expression of Wap65-2 with Wap65-1 and found their differential response to physiological stressors, such as low and high water temperature and bacterial challenge.

## Materials and methods

### Source of semen, blood, and tissues

For Wap65-2 isolation, semen was collected from mature common carp (*Cyprinus carpio* L.; Lithuanian strain B, weight 5 ± 2 kg, age 6–7 years) maintained at the Polish Academy of Sciences, Institute of Ichthyobiology and Aquaculture in Gołysz, Poland (experimental procedure allowance number: 32/2019). For the immunologic cross-reactivity study, semen of barbel (*Barbus barbus* L.; weight 224 ± 112 g, age 3 years), dace (*Leuciscus leuciscus* L.; weight 180 ± 60 g, age 3 years), chub (*Squalius cephalus* L.; weight 160 ± 40 g, age 3 years), burbot (*Lota lota* L.; weight 1.12–1.16 kg, age 4 years), grayling (*Thymallus thymallus* L.; weight 0.9–1.1 kg, age 3 years), rainbow trout (*Oncorhynchus mykiss* Walb.; weight 1.1–1.3 kg, age 3 years), ide (*Leuciscus idus* L.; weight 290 ± 140 g, age 3 years), asp (*Leuciscus aspius* L.; weight 2.1–3.2 kg, age 3 years), Siberian sturgeon (*Acipenser baerii* Brandt, 1869; weight 4–8 kg, age 6–8 years) was collected from mature fish raised in the Department of Lake and River Fishery of the University of Warmia and Mazury in Olsztyn (UWM), Rutki Salmonid Research Laboratory, Institute of Inland Fisheries in Olsztyn, and the Dgal Aquaculture Facility of the Inland Fisheries Institute in Olsztyn, Poland. Prior to milt collection, the fish were anesthetized using tricaine methane sulphonate (MS-222, Pharmaq Ltd., UK; 0.15 g L^−1^). Semen samples were centrifuged at 8000 × *g* at 4 °C for 10 min, followed by a centrifugation of supernatants at 10 000 × *g* at 4 °C for 10 min to obtain seminal plasma. For the temperature acclimation experiment, common carp males (weight 1324 ± 511 g, age 3+ years) were obtained from the Gosławice Fishery Farm and transported to the Department of Ichthyology and Aquaculture, UWM in Olsztyn (experimental procedure allowance number: 35/2017). The bacterial challenge experiment was performed on carp R3xR8 (weight 293 ± 112 g, age 2–3 years) obtained from Institute of Ichthyobiology and Aquaculture in Gołysz and transported to the Department of Evolutionary Immunology, Jagiellonian University, Krakow (experimental procedure allowance number: 49/2020). Tissue samples for mRNA expression and immunohistochemistry were obtained from fish killed by overdoses of MS-222 (0.5 g L^−1^), followed by decapitation. Samples were immediately covered with RNA preservation solution or fixed in Bouin solution.

### Purification of Wap65-2 from carp seminal plasma

A three-step isolation procedure developed in our laboratory was applied in order to obtain pure fractions of Wap65-2. Seminal plasma (13 mL) was adjusted to 1.25 M (NH_4_)_2_SO_4_, stored for 40 min at 4 °C, and then centrifuged at 10 000 × *g* for 10 min. The precipitate was discarded, and the supernatant was filtered through a 0.25-μm pore size syringe filter and further fractionated on X/K 16/10 of Phenyl Sepharose 6 Fast Flow (GE Healthcare, Uppsala, Sweden), a hydrophobic interaction (HIC) column that had been equilibrated with 1.25 M (NH_4_)_2_SO_4_ in 50 mM Tris–HCl (pH 7.6). Unbound proteins were eluted with four column volumes of the equilibrating buffer. Wap65-2 was bound under these conditions; bound proteins were eluted using a linearly decreasing concentration of (NH_4_)_2_SO_4_ (from 1.25 to 0 M) at a flow rate of 0.5 mL min^−1^ using an AKTA purifier system (GE Healthcare). The presence of Wap65-2 in the collected fractions was determined using electrophoretic methods [polyacrylamide gel electrophoresis (PAGE) and sodium dodecyl sulfate (SDS)-PAGE (details are provided as Additional file 1). Wap65-2 was eluted in the 20–15% of 1.25 M (NH_4_)_2_SO_4_ solution. The fractions of Wap65-2 (4–6 mL) were pooled, dialyzed for 24 h against 20 mM Tris–HCl (pH 7.6), and further fractionated by ion exchange chromatography (IEC) on a Q-Sepharose X/K 16/10 column (GE Healthcare) previously equilibrated with 20 mM Tris–HCl buffer (pH 7.6). After elution of unbound proteins, bound Wap65-2 was eluted using an increasing linear gradient of NaCl (from 0.00 to 0.35 M) in the same buffer over 135 min at a flow rate of 1 mL min^−1^. Protein fractions containing Wap65-2 were pooled, lyophilized, and subjected to preparative electrophoresis (PE; Mini Prep Cell, Bio-Rad, Hercules, California, USA) at an elution rate of 0.25 mL min^−1^. Electrophoresis was run in a 10% acrylamide gel (height 4.5 cm) at 500 V, 5 mA, and 4 °C. Wap65-2 fractions were dialyzed and then lyophilized for further analysis. Purification efficiency was monitored by Coomassie Brilliant Blue stain (CBB R-250) of PAGE and SDS-PAGE, according to the method of Laemmli [33], using a SE 250 vertical Mighty Small II electrophoresis system (GE Healthcare).

### Identification of Wap65-2

N-terminal protein sequence analysis was performed at BioCentrum (Kraków, Poland). The sequentially detached phenylthiohydantoin derivatives of amino acids were identified using the Procise 491 (Applied Biosystems, Warrington, Cheshire, UK) automatic sequence analysis system according to the manufacturer’s instructions. Details are provided in Additional file 1.

In-gel digestion and identification by matrix-assisted laser desorption/ionization time-of-flight/time-of-flight (MALDI TOF/TOF) mass spectrometry are provided in detail in Additional file 1. Mass spectra were acquired in the range of 700–3500 m/z using a MALDI-TOF Autoflex speed TOF/TOF mass spectrometer equipped with a Smartbeam II laser (355 nm; Bruker Daltonics, Bremen, Germany). The database search criteria were as follows: enzyme—trypsin; fixed modification—carbamidomethylation (C); variable modifications—methionine oxidation (M) peptide mass tolerance of 50 ppm, fragment mass tolerance of 0.7 Da; and one missed cleavage allowed. The search results were filtered using a significance threshold of p < 0.05 and a MASCOT ion score cutoff ≥ 30.

### Characterization of the physicochemical properties of Wap65-2

Details of the determination of the molecular weight and isoelectric point (pI), glycan moiety, enzymatic deglycosylation and interaction with lectins and phosphoprotein detection are provided in Additional file 4.

### Production and purification of monospecific polyclonal antibodies against Wap65-2

Polyclonal antibodies against the purified Wap65-2 were raised in rabbits. Immunization was performed as described by Wojtczak et al. [34]. The polyclonal anti-carp Wap65-2 IgG was purified from rabbit serum using a 1 mL HiTrap Protein A HP (GE Healthcare) affinity column [34]. Monospecific antibodies are antibodies purified by affinity purification with immobilized antigen as affinity ligand according to the procedure developed in our laboratory [35]. To obtain monospecific anti-Wap65-2 antibodies, fractions containing total IgG collected after Protein A chromatography were pooled and purified by affinity chromatography with immobilized Wap65-2 as a ligand [36]. This allowed the separation of anti-Wap65-2 IgG from a mixture of rabbit serum IgG (details in Additional file 1).

### Cross-reactivity between anti-Wap65-2 antibodies and seminal plasma of different fish species

Polyclonal antibodies against Wap65-2 were tested in order to detect this protein in the seminal plasma of barbel, dace, chub, turbot, grayling, rainbow trout, ide, asp, and Siberian sturgeon. Aliquots of seminal plasma (40 μg of protein) were separated by 1D SDS-PAGE. The western blot was performed as described by Dietrich et al. [31]. Polyclonal antibodies against Wap65-2 were diluted with Tris-buffered saline and 0.1% Tween 20 (TBS-T) at a ratio of 1:40,000. Additional details of the western blot procedure are provided in Additional file 1.

### Immunohistochemical localization of Wap65-2 in carp reproductive system and liver

The procedures used for immunohistochemistry were similar to those reported by Bilinska et al. [37]. Briefly, slices were immersed in 10 mM citrate buffer (pH 6.0) and heated (7 min, 650 W). Nonspecific staining was blocked with 0.3% hydrogen peroxide (H_2_O_2_) in methanol for 15 min, to inhibit endogenous peroxidase activity; and with 5% normal goat serum for 30 min at room temperature, to block nonspecific binding sites. Immunostaining was performed using carp monospecific polyclonal antibodies against Wap65-2 of carp seminal plasma (dilution 1:1000) followed by anti-rabbit IgG, and avidin biotinylated horseradish peroxidase complex (ABC/HRP) visualized by 3,3′-diaminobenzidine tetrachloride (DAB). Further details of the procedure are provided in Additional file 1. All immunohistochemical experiments were repeated at least three times. Control sections included omission of the primary antibody and substitution by pre-immune goat serum.

### Cloning and sequencing of Wap65-2 and sequence analysis

The full coding sequence of common carp Wap65-2 was obtained using several sets of primers (listed in Additional file 2) designed on the basis of the carp genomic sequence encoding a predicted hemopexin-like gene (LOC109051863 GenBank ID: XM_019069345) and two expressed sequence tags (GenBank ID: JZ198074; EX883418) from common carp and predicted zebrafish protein zgc:152945 (GenBank ID: XM_005173448). Sequences were aligned with Clustal Omega; primers were designed with Primer3. PCR was run using the KAPA2G Robust Hot Start Polymerase PCR Kit (Sigma-Aldrich, St. Louis, MO, USA) and 100 ng of pooled cDNA from carp liver as a template. The thermal profile included an initial denaturation step at 95 °C for 3 min, followed by 30 cycles of 95 °C for 30 s, 55 °C for 60 s, and 72 °C for 45 s, and a final extension step at 72 °C for 7 min. PCR was performed using an Eppendorf Mastercycler Gradient (Eppendorf, Hamburg, Germany). The PCR products were molecularly cloned as described earlier [27] and sequenced at LGC Genomics (Berlin, Germany).

The putative signal peptide was determined with SignalP 4.1 Server. The theoretical molecular mass and pI were calculated from the protein sequence with the ProtParamToll program. The posttranslational modifications (PTMs) *N*- and *O*-glycosylation sites were predicted with NetNGlyc 1.0 Server and NetOGlyc 3.1 Server, and the phosphorylation sites were predicted with NetPhos 2.0 Server. A phylogenetic analysis was performed using a PhyML 3.0 Approximate Likelihood Ratio Test: SH-like with tree rendering with TreeDyn 198.3. The protein homology/analogy recognition engine version 2.0 (Phyre2) was used for three-dimensional (3D) protein structure prediction. A multiple sequence alignment was generated by the ClustalW tool.

### Thermal treatments

After transport, fish were placed in tanks (1200 L; pH 7.2–7.8; oxygen content > 90%) at 18 °C for 1 week. To analyze the effect of water temperature on the expression of both Wap65 genes, fish were randomly separated into two groups: One group (n = 6) was acclimated to 10 °C while the second (n = 6) was acclimated to 30 °C for 5 weeks. The acclimation period and experimental design was determined in reference to Watabe et al. [6, 38] and Kinoshita et al. [9]. After thermal treatment, individual testes and spermatic ducts were collected in RNA preserving solution (70% ammonium sulphate in 25 mM sodium citrate and 20 mM EDTA) and stored at − 80 °C until further analysis.

### *Aeromonas salmonicida* infection

Reproductively mature male common carp were kept at 25 °C and infected by intraperitoneal injection with 200 µL of phosphate-buffered saline (PBS) containing 5 × 10^7^ colony-forming units (CFU)/mL of *A. salmonicida* (isolate H001) (n = 8). Control animals were injected with 200 µL sterile PBS (n = 8). Injections were performed under analgesia with 0.15 g L^−1^ MS222. Two days post-injection, samples from testis, spleen, kidney, and liver were collected, placed in RNA preserving solution, and stored at − 80 °C until further analysis.

### qPCR analysis of gene expression

Total RNA was isolated from the 20 mg of the tissues using the TRI reagent (Sigma-Aldrich). The quantity and quality of total RNA were assessed using a NanoDrop 1000 spectrophotometer (Thermo Fisher Scientific, Waltham, MA, USA) and agarose gel electrophoresis. Due to low-quality RNA obtained from the testis of two control fish, the cDNA was generated for n = 6 control and n = 8 *A. salmonicida*-infected individuals. The total RNA (200 ng) was treated with 1 U DNAse I (Thermo Fisher Scientific) and transcribed to cDNA through random hexamer and oligo(dT)18 primers using 100 U Maxima Reverse Transcriptase (Thermo Fisher Scientific). A non-reverse transcriptase control for genomic DNA contamination was included in the analysis for each sample. cDNA samples were diluted 1:40 with nuclease-free water (Thermo Fisher Scientific) before qPCR analysis. Plasmid-based quantification using SYBR Green intercalating dye qPCR was performed on duplicate samples, using Maxima SYBR Green/ROX qPCR Master Mix (Thermo Fisher Scientific). The sequences of the primers are listed in Additional file 2. The expression of analyzed genes was assessed relative to the geometric mean of two reference genes that encode 40S ribosomal protein S11 (40S) and elongation factor 1 alpha (*ef1a*). The results are presented as normalized copies per 100 000 copies of reference genes. For tissue libraries, the cDNA obtained in earlier research was used [39].

### Statistical analyses

The gene expression results were logarithmically transformed, namely log_10_(x). Data normality was evaluated with the Shapiro–Wilk test, and equality of variances was tested with the F-test. After passing both tests, the data were subjected to two-way (effect of temperature acclimation and *A. salmonicida* infection) or one-way (*A. salmonicida* 16S rRNA gene expression) analysis of variance (ANOVA) with all pairwise multiple comparison procedures performed with Holm–Sidak method in Sigmaplot 12.5. The differences were recognized as statistically significant at p < 0.05.

## Results

### Purification of Wap65-2 from carp seminal plasma

**Figure 1:**
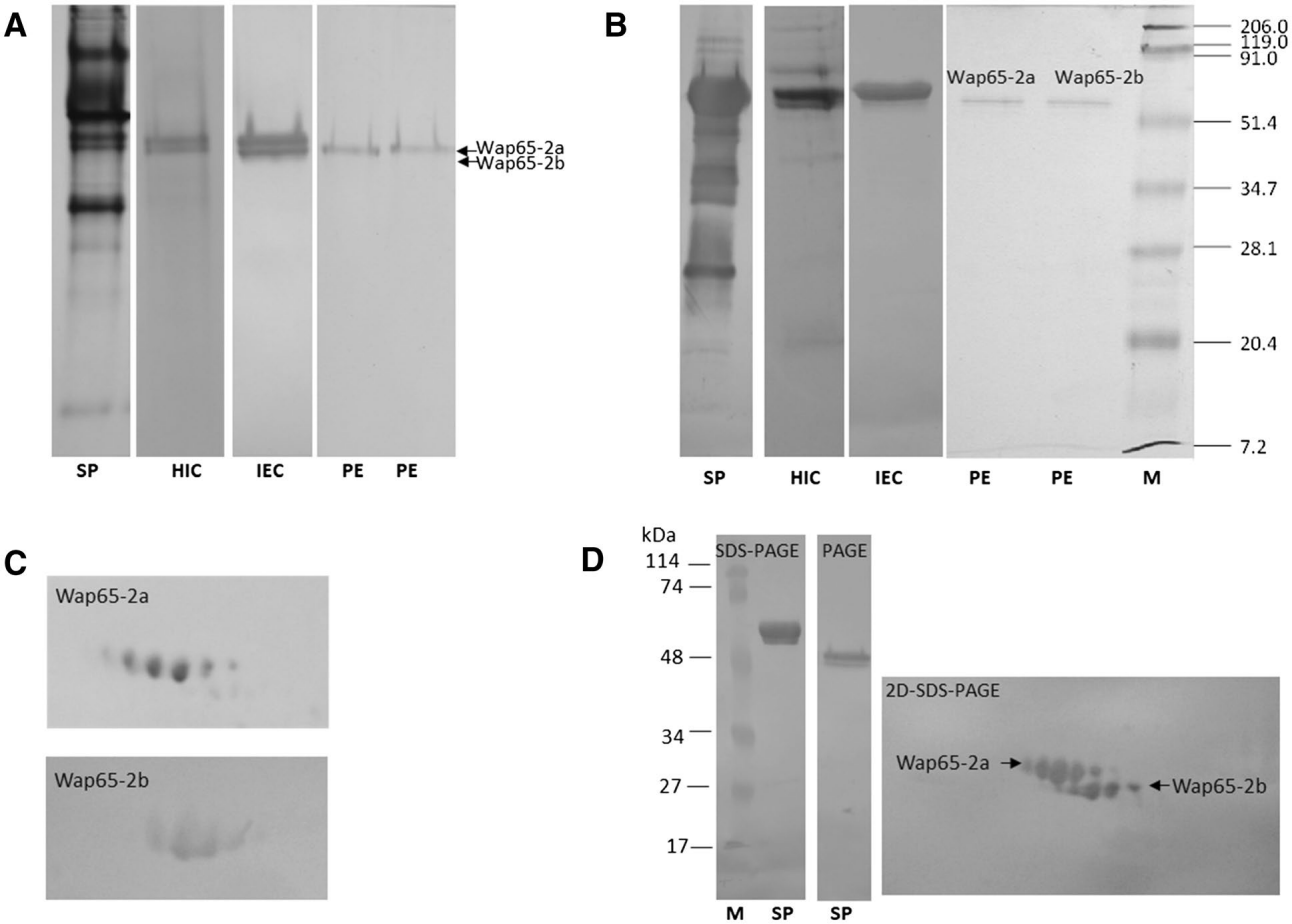
**Purification of the Wap65-2 from carp seminal plasma.** Lines: seminal plasma (SP) hydrophobic interaction (HIC) fraction; ion exchange chromatography (IEC) fraction, and fraction eluted after preparative electrophoresis (PE). Protein samples were analyzed by PAGE (**A**) and SDS-PAGE (**B**). Proteoforms of isolated Wap65-2a and Wap65-2b after 2-DE (**C**). Detection of Wap65-2 in carp seminal plasma after SDS-PAGE, native PAGE, and 2-DE using anti-Wap65-2 antibodies (**D**). Additional file 4 indicates which part of the corresponding 2-DE of seminal plasma is presented on the blot (**D**).

Using Phenyl Sepharose HIC chromatography, we separated Wap65-2, which comprised two bands (Wap65-2a, Wap65-2b), from most carp seminal plasma proteins (Figures 1A, B). SDS-PAGE revealed that the fraction containing Wap65-2 after HIC were contaminated; therefore, IEC was applied to obtain pure Wap65-2 (two bands). Final separation of Wap65-2a and Wap65-2b was achieved using PE. The 2-DE of pure Wap65-2a and Wap65-2b preparations revealed six proteoforms of Wap65-2a and four proteoforms of Wap65-2b (Figure 1C). These proteoforms were also detected in carp seminal plasma using anti-Wap65-2 antibodies (Figure 1D). To confirm that the detected spots in seminal plasma indeed corresponded to Wap65-2, all spots related to the blot were identified by mass spectrometry MALDI-TOF/TOF (Additional file 3).

### Identification of Wap65-2

**Figure 2:**
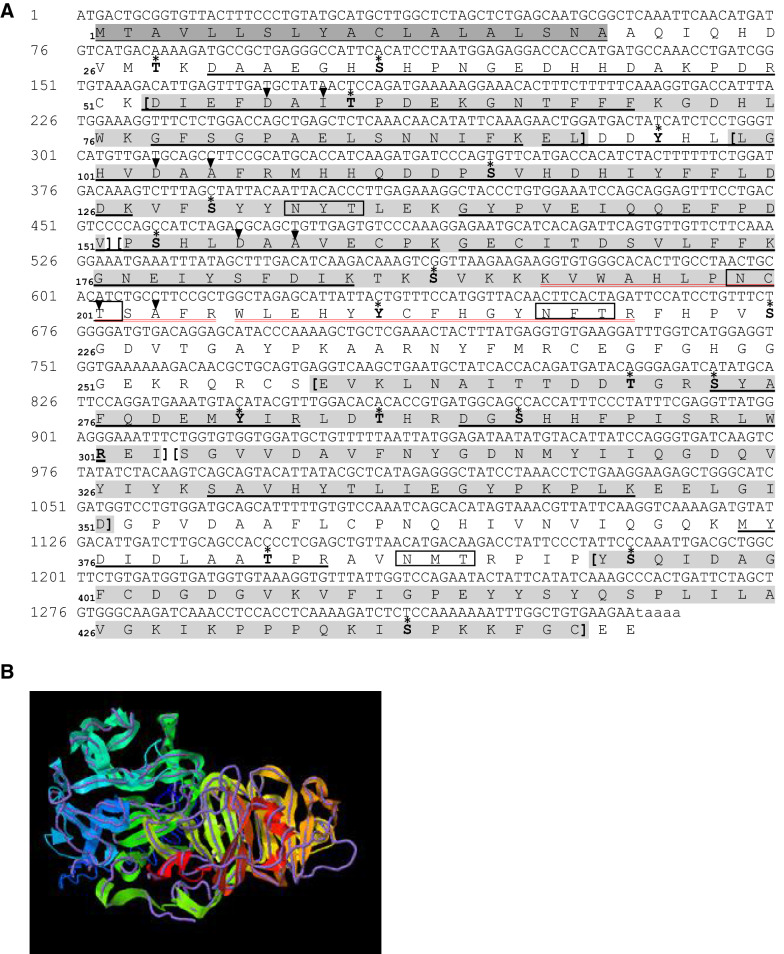
**cDNA and deduced amino acid sequence of carp Wap65-2 (A) and predicted tertiary structure of carp Wap65-2 with rabbit hemopexin (B).** The signal peptide sequence is shaded in dark grey and the six hemopexin-like repeats in the sequence are shaded in light grey. The inverted dark triangles indicate metal ion binding sites. Potential *N*-linked glycosylation sites are boxed, and predicted phosphorylation sites are denoted with stars. Peptides identified by mass spectrometry are underlined. Peptides identified after *N*-glycosylation are double underlined in red. Wap65-2 is shown as a colored cartoon, while the structural analog (rabbit hemopexin) is displayed using a backbone trace.

N-terminal Edman sequencing revealed the blockage of the Wap65-2a preparation and allowed the identification of only eight amino acids (DAAEGHSH) for the Wap65-2b preparation. Therefore, Wap65-2a and Wap65-2b were subjected to in-gel trypsin digestion and MALDI-TOF/TOF mass spectrometry. Wap65-2a was identified based on mass spectrometry identification of 12 unique peptides showing 60% sequence coverage with PREDICTED: hemopexin-like [*Cyprinus carpio*], and Wap65-2b based on 14 unique peptides showing 58% sequence coverage with PREDICTED: hemopexin-like [*Cyprinus carpio*] (Table 1). The Wap65-2 amino acid sequence was deduced from the Wap65-2 cDNA sequence obtained from carp liver in the present work (Figure 2).

**Table 1:** Characteristics of the Wap65-2 peptides identified by mass spectrometry MALDI-TOF/TOF

Protein accession, description	Sequence coverage	Mascot score	Precursor mass		Peptide score	Peptide sequence
			Observed	Theoretical		
Wap65-2a						
XP_018924890	60	692	1392.6594	1391.6456	51	K.DIEFDAITPDEK.G
PREDICTED: hemopexin-like [*Cyprinus carpio*]			860.4074	859.4229	61	K.GNTFFFK.G
			1480.7651	1479.7358	39	K.GFSGPAELSNNIFK.E
			1870.9130	1869.9010	138	K.ELDDYHLLGHVDAAFR.M
			2496.1309	2495.0965	163	R.MHHQDDPSVHDHIYFFLDDK.V
			2825.2896	2824.3378	165	K.GYPVEIQQEFPDVPSHLDAAVECPK.G
			1415.7103	1414.6803	38	K.GECITDSVLFFK.G
			1185.5932	1184.5713	64	K.GNEIYSFDIK.T
			1422.6446	1421.6285	66	R.SYAFQDEMYIR.L
			1152.5800	1151.5472	63	R.DGSHHFPISR.L
			1816.0287	1814.9930	43	K.SAVHYTLIEGYPKPLK.E
			1265.6452	1264.6122	78	K.MYDIDLAATPR.A
			1558.7466	1557.7511	89	K.VWAHLP**N**CTSAFR.W^a^
			2093.8218	2091.9050	114	R.WLEHYYCFHGY**N**FTR.F^a^
Wap65-2b						
XP_018924890	58	752	2292.9663	2290.9700	123	K.DAAEGHSHPNGEDHHDAKPDR.C
PREDICTED: hemopexin-like [*Cyprinus carpio*]			1392.6594	1391.6456	51	K.DIEFDAITPDEK.G
			860.4074	859.4229	61	K.GNTFFFK.G
			1480.7651	1479.7358	39	K.GFSGPAELSNNIFK.E
			1870.9130	1869.9010	138	K.ELDDYHLLGHVDAAFR.M
			2496.1309	2495.0965	163	R.MHHQDDPSVHDHIYFFLDDK.V
			2825.2896	2824.3378	165	K.GYPVEIQQEFPDVPSHLDAAVECPK.G
			1415.7103	1414.6803	38	K.GECITDSVLFFK.G
			1185.5932	1184.5713	64	K.GNEIYSFDIK.T
			1422.6446	1421.6285	66	R.SYAFQDEMYIR.L
			1152.5800	1151.5472	63	R.DGSHHFPISR.L
			1816.0287	1814.9930	43	K.SAVHYTLIEGYPKPLK.E
			1265.6452	1264.6122	78	K.MYDIDLAATPR.A
			1558.7466	1557.7511	46	K.VWAHLP**N**CTSAFR.W
			2093.8218	2091.9050	114	R.WLEHYYCFHGY**N**FTR.F^a^

^a^Peptides identified after deglycosylation, **N**–N-linked glycosylation sites.

### Identification and characterization of Wap65-2 cDNA and amino acid sequence

The obtained Wap65-2 cDNA and amino acid sequence are shown in Figure 2A; it has been published under GenBank accession no. ATP66527.1. The cDNA clone contains an open reading frame of 1,335 base pairs (bp) that encodes 445 amino acids (Figure 2). The predicted signal peptide cleavage site is located between amino acids 19 and 20 (SNA-AQ), resulting in a 426-residue protein. The calculated molecular mass is 48.58 kDa, with a theoretical pI of 5.71. There are 18 potential phosphorylation sites (10 at serine residues; five at threonine residues and three at tyrosine residues). There are four potential *N*-glycosylation sites (marked in the frame) at positions 133, 199, 217, and 387 but no potential *O*-glycosylation sites. The 3D structural analysis of carp Wap65-2 revealed that the best template in the database using X-ray diffraction was Hpx from the rabbit *Oryctolagus cuniculus* (C-score of 2.45 and a TM-score of 0.85; Figure 2B).

**Figure 3:**
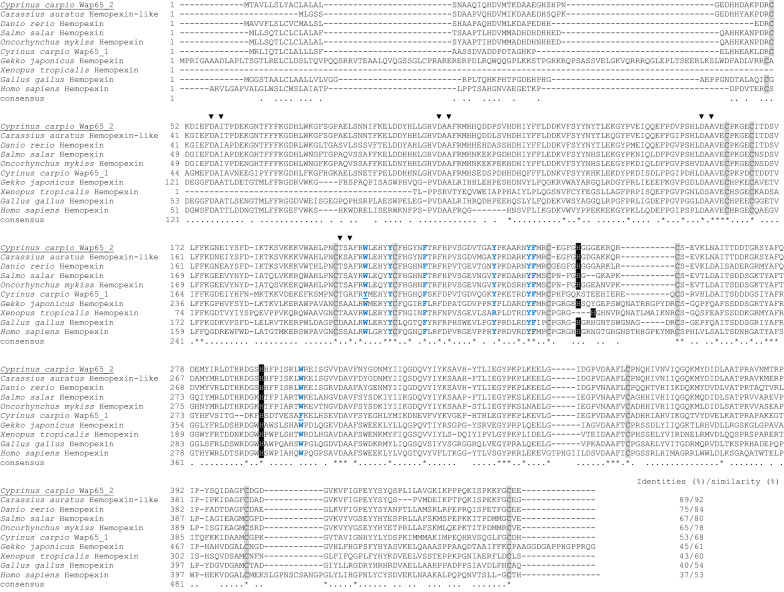
**Multiple amino acid alignment comparing Wap65-2 sequences from carp with other vertebrates.** Asterisks mark the identical amino acids in all sequences. Conserved histidine residues (His260 and His292), crucial for heme binding, are highlighted in dark shadowed boxes; conserved 10 cysteine residues, essential for structural integrity forming disulfide bridges, are presented in light shadowed boxes. Metal ion binding sites are marked with black triangles (NCBI GenBank accession numbers of the utilized sequences are listed in Additional file 5).

The complete amino acid sequence of carp Wap65-2 displayed the highest (99%) identity with PREDICTED: hemopexin-like [*Cyprinus carpio*] (XP_018924890.1). The alignment among Wap65-2 with other sequences revealed high homology from 75 to 89% identity with Wap65-2 (hemopexin-like) of cyprinids and 65–67% with salmonids and moderate 45%, 43%, 40% and 37% homology to gecko (*Gekko japonicas;* XP_015280493.1), frog (*Xenopus tropicalis*; XP_002944396.2), chicken (*Gallus gallus*; XP_015136422.1), and human (AAA52704.1) Hpx, respectively. The identity and similarity scores between Wap65-2 and Wap65-1 were 53 and 68%, respectively. Wap65-2 have common features typical for the protein structure of mammalian Hpx including conserved 10 cysteine residues that form disulfide bridges, seven conserved aromatic residues, two conserved histidine residues crucial for heme binding, and metal binding sites (Figure 3).

**Figure 4:**
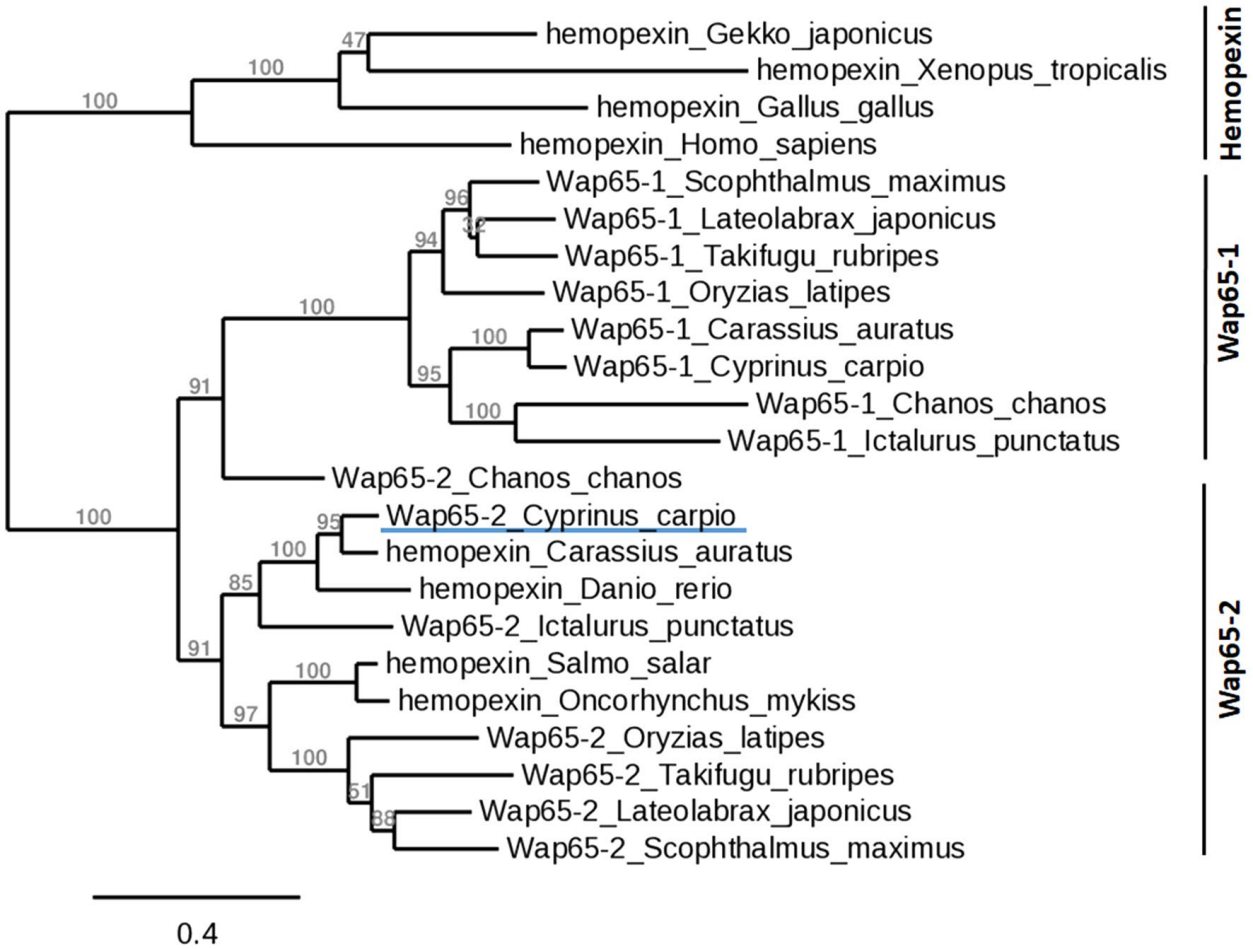
**Phylogenetic analysis of the complete amino acid sequences of carp Wap65-2 and different species.** A phylogenetic analysis was performed using a PhyML 3.0 Approximate Likelihood Ratio Test: SH-like with tree rendering with TreeDyn 198.3. (NCBI GenBank accession numbers of the utilized sequences are listed in Additional file 5).

The phylogenetic tree revealed that carp Wap65-2 was grouped in a different clade from carp Wap65-1 (BAB60809.1), although both forms clustered with other teleost species (Figure 4, Additional file 5). On the other hand, Hpxs from other vertebrates formed a paraphyletic group.

### Determination of molecular weight and pI

The molecular mass of two forms of Wap65-2 measured by mass spectrometry were 64.5 kDa and 63.4 kDa for Wap65-a and Wap65-b, respectively. Analysis via 2-DE revealed six proteoforms of Wap65-2a with a pI from 5.05 to 5.23 and four proteoforms of Wap65-2b with a pI from 5.18 to 5.30 (Figure 1C, Additional file 6). The term “proteoform” describe “all of the different molecular forms of a protein product arising from a single gene” [40]. To confirm that the detected spots indeed corresponded to Wap65-2, all spots were identified by MALDI-TOF/TOF mass spectrometry (Additional file 3).

### Interaction with lectins and enzymatic deglycosylation

**Figure 5:**
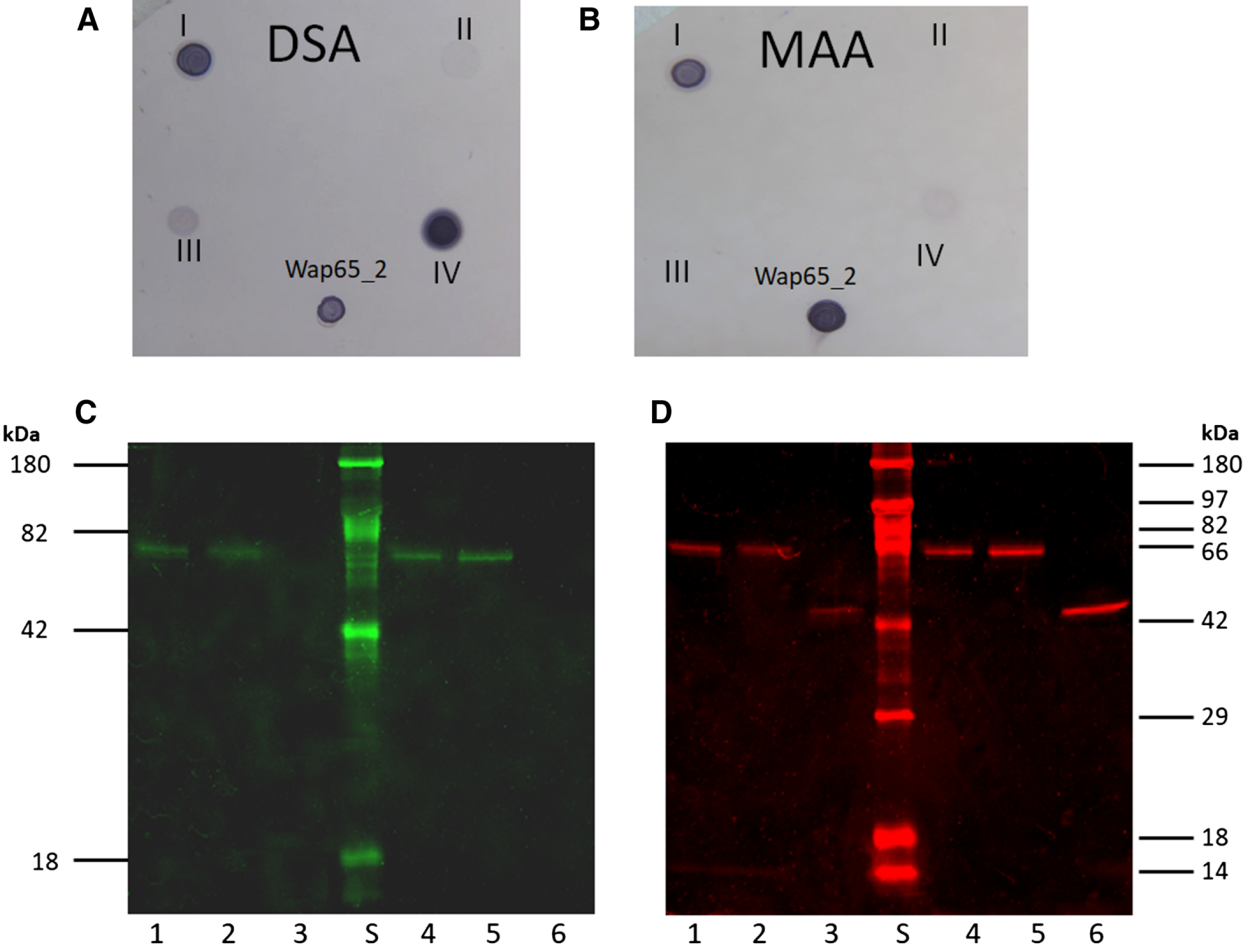
**Interaction with lectins (A, B) and deglycosylation of carp seminal plasma Wap65-2a and Wap65-2b (C, D).** *N*-Glycosidase F (PNGase F) (+N) and *O*-glycosidase (+O). Staining for positive *Datura stramonium* agglutinin (DSA; **A**) and *Maackia amurensis* agglutinin (MAA; **B**) affinities of Wap65-2 (I—fetuin, II—carboxypeptidase Y, III—transferrin, and IV—asialofetuin). Fluorescent staining for glycoproteins using Pro-Q Emerald 300 (**C**) and for proteins using SYPRO Ruby (**D**). Lane identities: S—molecular mass marker, 1—Wap65-2a (control), 2—Wap65-2a incubated with *O*-glycosidase, 3—Wap65-2a incubated with *N*-glycosidase, 4—Wap65-2b (control), 5—Wap65-2b incubated with *O*-glycosidase, and 6—Wap65-2b incubated with *N*-glycosidase.

There were positive reactions of the two Wap65-2a and Wap65-2b with digoxigenin-labeled lectins: *Maackia amurensis* agglutinin (MAA) and *Datura stramonium* agglutinin (DSA) (Figure 5A, B). The strong positive reaction with MAA and DSA indicate the presence of sialic acid terminally linked to galactose mostly via α(2–3) and the presence of d-galactose-β(1–4)-*N*-acetylglucosamine. The negative reaction with *Galanthus nivalis* agglutinin (GNA) indicates the lack of a terminally linked mannose, a feature that excludes the presence of high mannose or hybrid-type carbohydrate chains. Carp seminal plasma Wap65-2 did not contain the *O*-glycosidically linked carbohydrate chains, as evidenced by the negative reaction with peanut agglutinin (PNA).

Electrophoretic analysis of both Wap65-2a and Wap65-2b after deglycosylation with peptide-*N*-Glycosidase F (PNGase F) revealed decreases in molecular mass from 64.49 ± 0.7 kDa to 45.7 ± 0.8 kDa and from 63.38 ± 0.6 to 46.28 ± 0.4, respectively (Figure 5C, D). Thus, 29% of Wap65-2a and 27% of Wap65-2b are carbohydrate moieties. After incubation with *O*-glycosidase, there were no differences in electrophoretic migration rates of Wap65-2a and Wap65-2b compared to the control samples.

By comparing the mass spectrometry results obtained with Wap65-2 and the deglycosylated Wap65-2, we identified two additional peptides (_192_KVWAHLPNCTSAFRW_206_ and _205_RWLEHYYCFHGYNFTRF_221_) for Wap65-2a and one peptide (_205_RWLEHYYCFHGYNFTRF_221_) for Wap65-2b. Asparagine at position 199 and 217 was predicted to be *N*-glycosylated with NetNGlyc (Table 1, Figure 2A).

### Phosphoprotein detection

Phosphoprotein staining using Pro-Q Diamond Phosphoprotein solution was positive for all Wap65-2a and Wap65-2b proteoforms (Additional file 7) indicating presence of phosphate moiety in their structures. There was a very weak reaction for Wap65-2 after dephosphorylation.

### Cross-reactivity between anti-Wap65-2 antibodies and seminal plasma of different fish species

**Figure 6:**
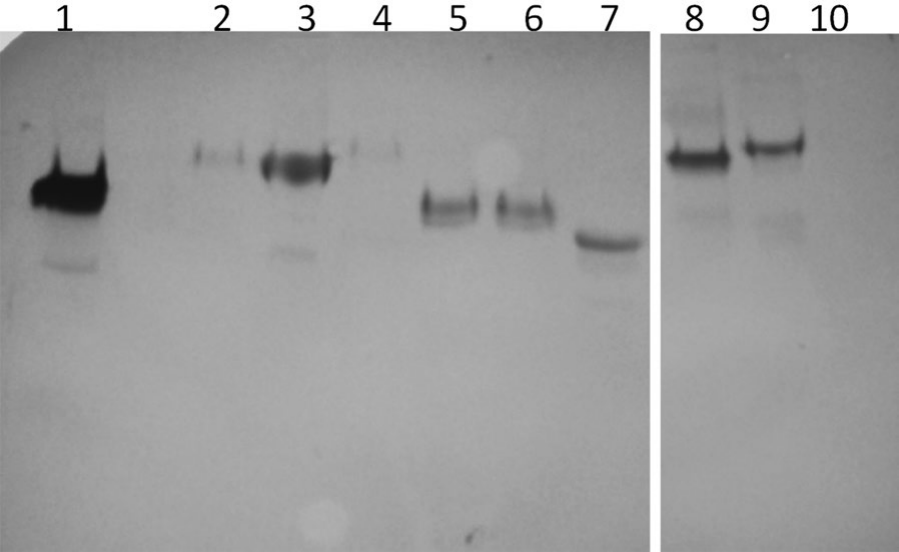
**Western blot analysis of Wap65-2 in seminal plasma of fish species.** 1—carp, 2—barbel, 3—dace, 4—chub, 5—burbot, 6—grayling, 7—rainbow trout, 8—ide, 9—asp, and 10—sturgeon. Seminal plasma proteins were separated using 1D-SDS-PAGE.

SDS-PAGE and western blot analysis revealed that polyclonal antibodies against Wap65-2 reacted with seminal plasma from carp, dace, turbot, grayling, rainbow trout, ide, and asp. Furthermore, there was a weak reaction for seminal plasma of barbel and chub and no reaction for Siberian sturgeon seminal plasma (Figure 6).

### Immunohistochemical localization of Wap65-2 in the carp reproductive system and liver

**Figure 7:**
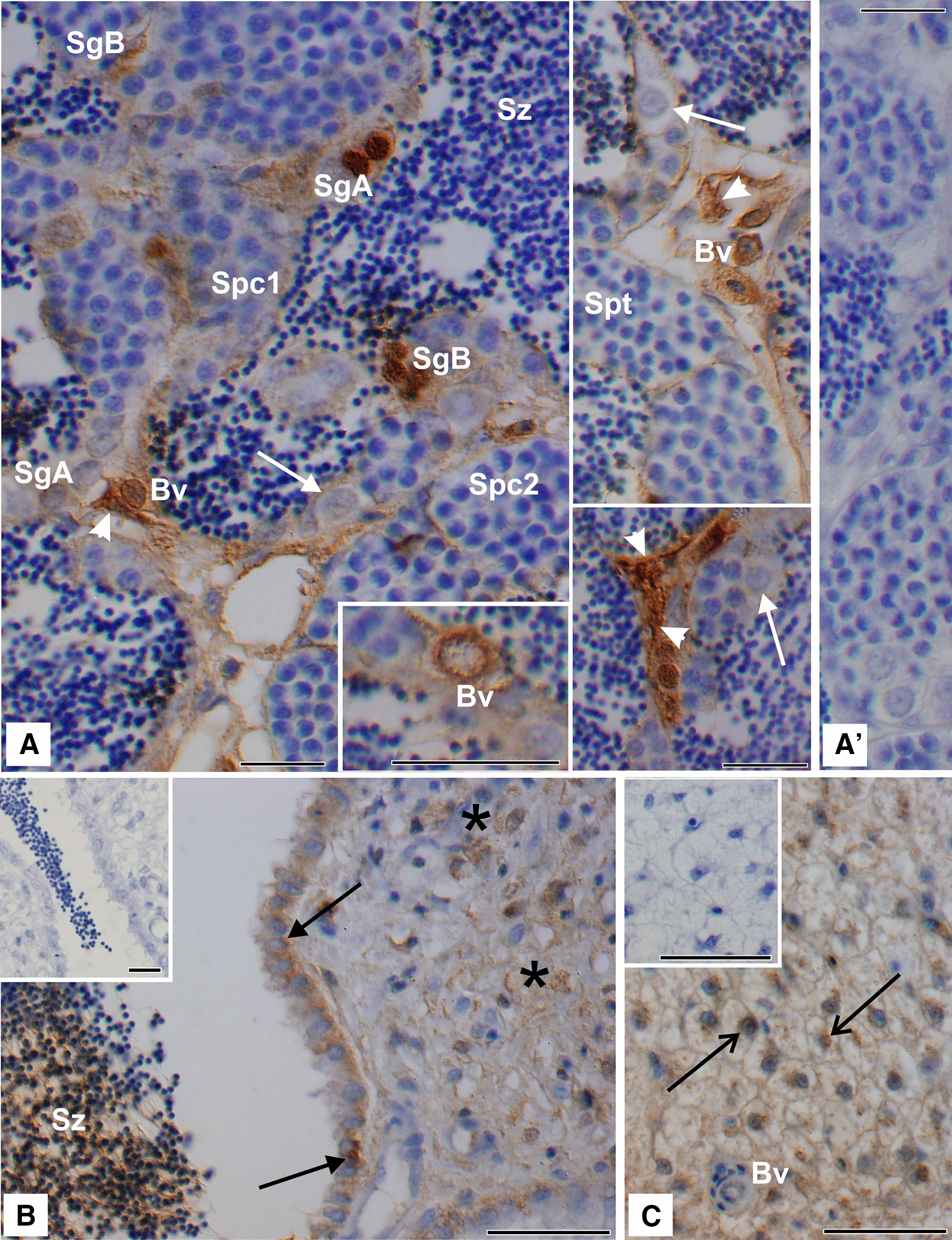
**Immunohistochemical localization of Wap65-2 in carp testis (A), spermatic duct (B), and liver (C).** Bars = 10 μm. **A** Strong signal for Wap65-2 is present in some cysts that contain spermatogonia A (SgA) and spermatogonia B (SgB), as well as in Leydig cells (arrowheads). At higher magnification (bottom image), note the strong signal around the epithelium of blood vessels (Bv). No immunopositive staining is observed in primary (Spc1) and secondary spermatocytes (Spc2), spermatids (Spt), and Sertoli cells (arrows). Immunonegative spermatozoa (Sz) are clearly visible. **B** In the spermatic duct, there is weak to moderate signal for Wap65-2 in columnar secretory cells (black arrows) and stromal cells (asterisks). Positive staining from luminal epithelium is visible near spermatozoa. **C** In the liver cells, there is a positive moderate signal for Wap65-2 localized to hepatocytes. Note the perinuclearly located signals (open arrows). Insets in **A**–**C** controls of testicular, spermatic duct, and liver cells when the primary antibody is omitted respectively.

Immunohistochemical analyses revealed positive staining, albeit with different intensities for Wap65-2 in carp testis, spermatic duct, and liver (Figure 7A–C). Strong signal was localized to some cysts containing spermatogonia A and spermatogonia B (early-late) and to interstitial Leydig cells. Of note, in spermatogonia A and B and Leydig cells, both the cytoplasm and the nuclei were immunopositive for Wap65-2. In contrast, primary and secondary spermatocytes, spermatids, and Sertoli cells displayed no Wap65-2 immunoreactivity (Figure 7A). Moreover, spermatozoa within the lumina of testicular lobules were immunonegative for the Wap65-2 (Figure 7A). Interestingly, the epithelium around blood vessels within the testis was strongly stained (Figure 7A). In the epithelium of the spermatic duct, columnar secretory cells surrounded by the connective tissue with stromal cells displayed weak to moderate immunoreactivity for the Wap65-2 protein (Figure 7B). A moderate positive reaction, visible in spermatic duct lumina near spermatozoa, may reflect the presence of the protein from the epithelial cell secretion (Figure 7B). In the liver, positive Wap65-2 signal was localized to the perinuclear region of hepatocytes (Figure 7C). In contrast to testicular blood vessels, the liver blood vessels displayed no positive Wap65-2 staining (Figure 7C). The control sections of testicular, spermatic duct, and liver tissue, in which the primary antibodies were omitted, did not exhibit any positive Wap65-2 staining (Figure 7A and insets in Figure 7B, C).

### mRNA expression profiles of Wap65-2 in carp tissues in relation to Wap65-1

**Figure 8:**
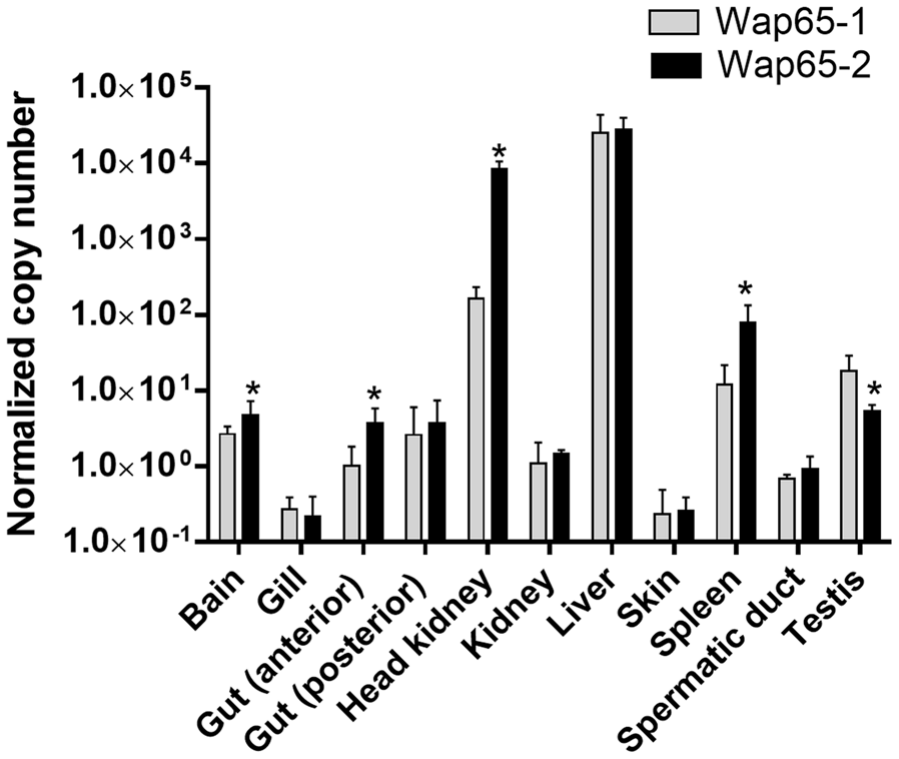
**Expression of carp Wap65-2 and Wap65-1 mRNA in carp tissues (n = 4).** The results are presented as normalized copies per 100 000 copies of reference genes 40S ribosomal protein S11 (40S) and elongation factor 1 alpha. Stars indicate differences between Wap65-1 and Wap65-2.

Both Wap65-2 and Wap65-1 genes were expressed in all examined carp tissues, including brain, gill, gut, kidney, liver, skin, spleen, spermatic duct, and testis (Figure 8). The highest gene expression for both Wap65-1 and Wap65-2 occurred in the liver; Wap65-2 was also highly expressed in the head kidney (Figure 8). Wap65-2 gene expression in liver was 5300 and 30 000 times higher than in the testis and spermatic duct, respectively, while its gene expression in testis was 5 times higher than in the spermatic duct. Wap65-2 gene expression was higher in the brain, gut, head kidney, and spleen compared with Wap65-1. By contrast, Wap65-1 was more highly expressed in the testis compared with Wap65-2.

### Effect of acclimation to cold and warm temperatures on Wap65s mRNA level in reproductive system

**Figure 9:**
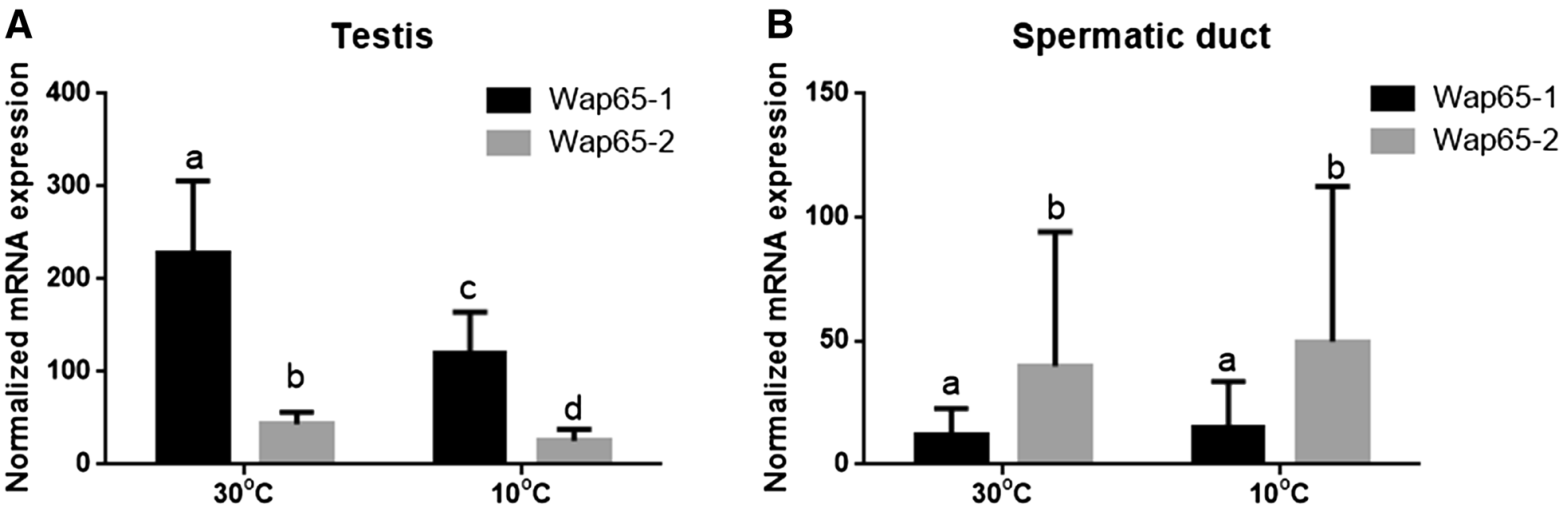
**mRNA expression of Wap65-1 and Wap65-2 in carp reproductive system [testis (A) and spermatic duct (B)] after temperature acclimation (n = 6 per condition).** The results are presented as normalized copies per 100 000 copies of reference genes 40S ribosomal protein S11 and elongation factor 1 alpha. The bars represent means ± standard deviation. Different letters indicate significant differences between groups.

Wap65-2 and Wap65-1 gene expression was higher in the testis of fish acclimated to 30 ˚C compared with 10 ˚C (Figure 9A). Similar differences were not found in spermatic duct (Figure 9B). In carp subjected to 30 ˚C or 10 ˚C, the testis gene expression of Wap65-1 was higher than Wap65-2, while in the spermatic duct, Wap65-2 was more highly expressed than Wap65-1 (Figure 9).

### Expression of Wap65-2 after *A. salmonicida* infection in relation to Wap65-1

**Figure 10:**
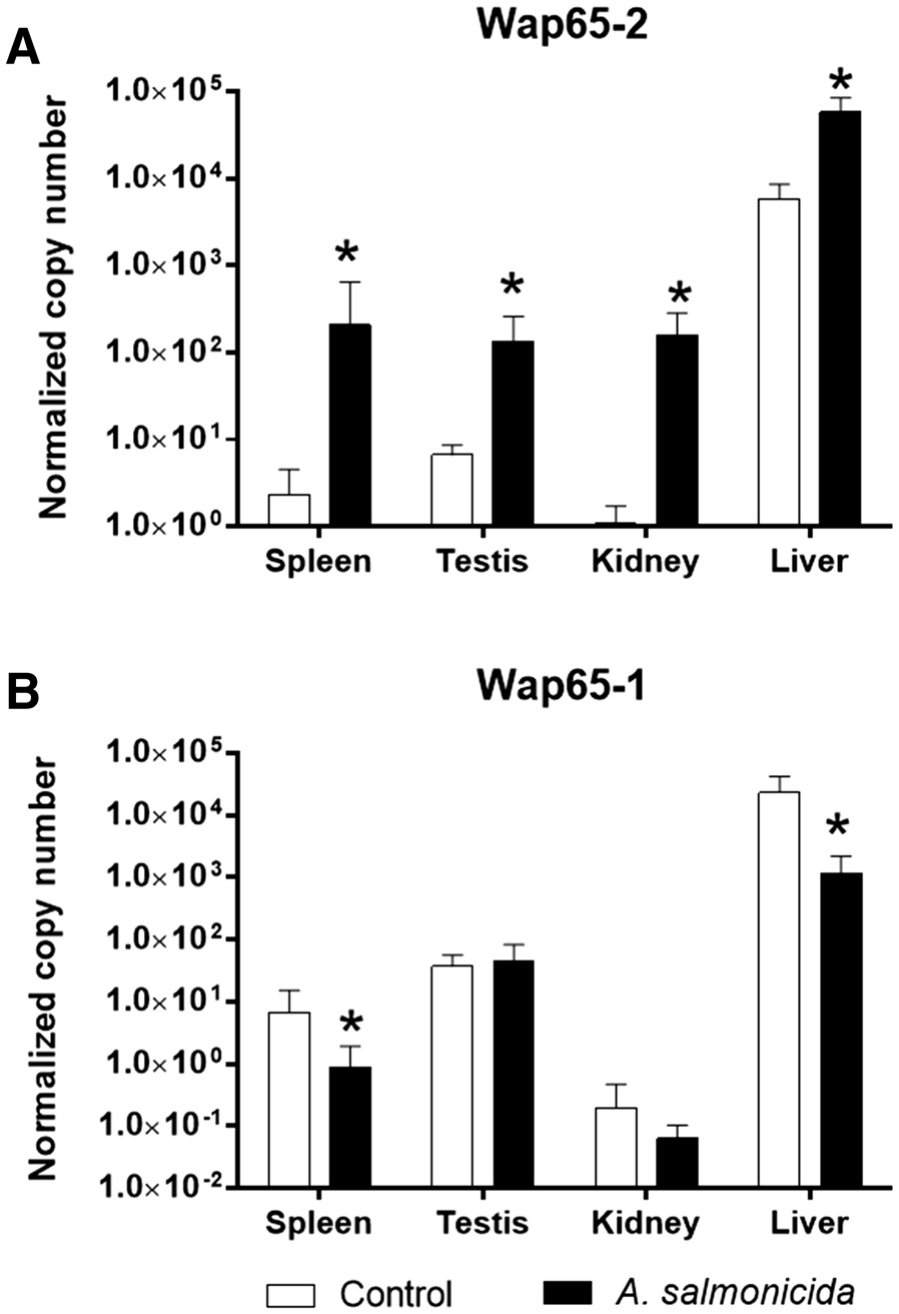
**Modulation of mRNA expression of Wap65-2 (A) and Wap65-1 (B) in tissues during bacterial challenge with** ***A. salmonicida*** **(n = 6 per condition).** The results are presented as normalized copies per 100 000 copies of reference genes 40S ribosomal protein S11 and elongation factor 1 alpha. The bars represent means ± standard deviation. Stars indicate significant differences between the control and treated group.

Infection with the gram-positive bacteria *A. salmonicida* led to elevated Wap65-2 mRNA expression in all examined tissues (Figure 10A): its level was 145 times higher in the kidney, 90 times higher in the spleen, 20 times higher in the testis, and 10 times higher in the liver after bacterial infection compared with the control. In contrast, Wap65-1 mRNA expression in infected fish was lower in the liver and spleen compared with the control fish (Figure 10B). The mRNA expression decreased 8- and 20-fold in spleen and liver, respectively. There were no differences in Wap65-1 gene expression in the testis and kidney.

### Expression of immune-related genes in carp tissue after bacterial challenge with A. salmonicida and quantification of bacterial 16S rRNA genes

**Figure 11:**
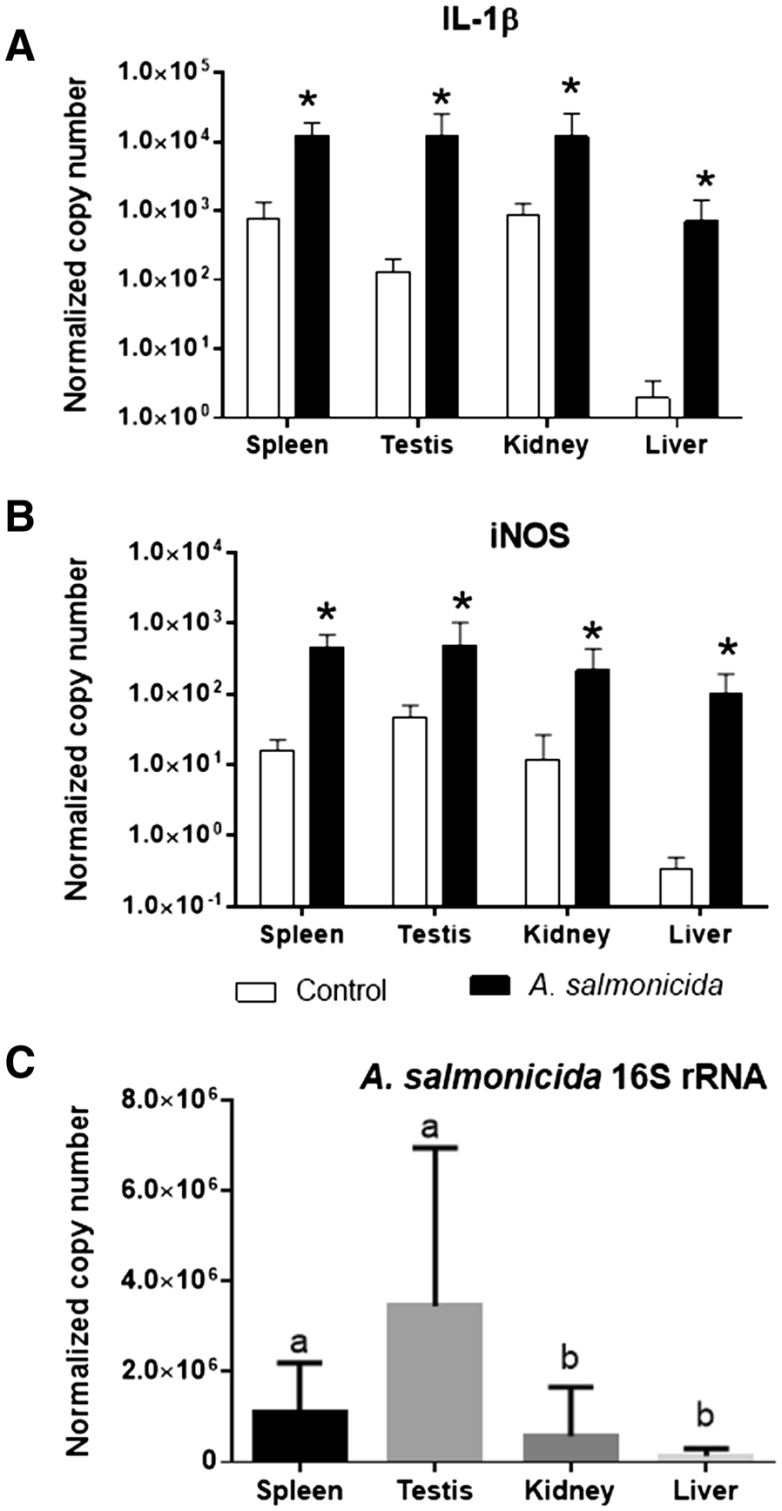
**Expression of immune-related genes and 16S rRNA in carp tissue after bacterial challenge with** ***A. salmonicida.*** IL-1βl—interleukin 1β (**A**), iNOS—inducible nitric oxide synthase (**B**). Quantification of *A. salmonicida* in tissues using 16S rRNA gene expression (**C**) (n = 6). The results are presented as normalized copies per 100 000 copies of reference genes 40S ribosomal protein S11 and elongation factor 1 alpha. The bars represent means ± standard deviation. Stars indicate significant differences between the control and treated group (**A**, **B**) while different letters indicate differences between tissues (**C**).

The mRNA expression of both interleukin 1β (IL-1β) and inducible nitric oxide synthase (iNOS) increased in all examined tissues after bacterial infection (Figure 11). Upon infection, IL-1β gene expression increased 356-fold, 93-fold, 17-fold, and 14-fold in the liver, testis, spleen and kidney, respectively, while iNOS gene expression increased 294-fold, 29-fold, 18-fold, and tenfold in the liver, spleen, kidney, and testis, respectively.

*Aeromonas salmonicida* in the tissues were quantified by 16S rRNA gene analysis using qPCR. *A. salmonicida* was determined in all tissues tested with the highest 16S rRNA gene expression in the testis, follow by spleen, kidney and liver (Figure 11C). 16S rRNA gene expression in the testis was 24 and 6 times higher than in the liver and kidney, respectively.

## Discussion

In this study, we identified and characterized a second isoform of Wap65, which branched into the Wap65-2 subgroup, in carp seminal plasma. We provided a full-length cDNA sequence of Wap65-2 with several highly conserved features of mammalian Hpx and substantial differences from the previously identified carp Wap65-1. Wap65-2 from carp seminal plasma comprised several proteoforms and corresponded to the major proteins of carp seminal plasma. There was high similarity of carp Wap65-2 with their counterparts in seminal plasma of other fish species. Wap65-2 mRNA was detected in all analyzed tissues, with predominant expression in the liver. Moreover, Wap65-2 protein was localized to spermatogonia, Leydig cells, the epithelium of blood vessels in the testis, and the epithelium of spermatic duct. For the first time, we demonstrated a differential response of Wap65-2 and Wap65-1 to physiological stressors, such as low and high water temperature and bacterial challenge within the fish reproductive system and demonstrated that bacterial infection triggers testicular immunity.

We developed an efficient method for the purification and isolation of two proteoforms of Wap65-2 from carp seminal plasma differing in electrophoretic mobility in SDS-PAGE; it comprises three steps: HIC, IEC, and PE. This method allowed us to obtain pure Wap65-2a and Wap65-2b preparations that we successfully used in all experiments to identify and characterize these proteins. When we finally identified Wap65-2, we found that hemin-agarose affinity chromatography can be potentially useful for its isolation; however, its success depends on the fish species [11, 21]. In our study, we confirmed the usefulness of hemin-agarose affinity chromatography for isolation of Wap65-2 from carp seminal plasma (Additional files 1, 8). This outcome strongly suggests that in future studies affinity chromatography can be used as an alternative or additional step for Wap65-2 isolation from carp seminal plasma.

The obtained carp Wap65-2 sequence shared the common structural features with Hpx of all vertebrate species. These features include two hemopexin domains, hemopexin repeats, four heme-binding sites, five out of six pairs of conserved cysteine residues essential for Hpx structural integrity, seven conserved aromatic residues that are invariable and define the heme pocket, and two critical conserved histidine residues assumed to serve as heme axial ligands (i.e., the formation of bis-histidyl heme iron complex) in mammalian Hpx [41]. Contrary to Wap65-2, carp Wap65-1 showed less similarity to Hpx because it only contains five out of seven conserved aromatic residues and one out of two conserved histidines. High structural and sequence homology of carp Wap65-2 to mammalian Hpx suggests that this form is more evolutionary conserved than Wap65-1, as previously reported for other teleost species [11, 21]. Moreover, our phylogenetic analysis grouped Wap65-2 with its respective orthologs from other fish species, showing a separate clade from previously identified carp Wap65-1, with closed phylogenetic relations to cyprinid species in each clade. The presence of two different Wap65 forms seems to be specific for fish. For other vertebrates, only Hpx resembling Wap65-2 has been identified; therefore, Wap65-1 seems to be a fish-specific protein.

Our study indicated differences in biochemical characteristics, including molecular mass, pI, glycosylation status, and the N-terminus, between Wap65-2a and Wap65-2b. The determined molecular mass of native Wap65-2 from carp seminal plasma (64.49 kDa for Wap65-2a and 63.38 kDa for Wap65-2b) was markedly higher than the calculated mass of the mature protein (47.43 kDa). This difference could be explained by high content of carbohydrate moieties (up to 29%) in the Wap65-2 structure, a feature that is a typical characteristic of Hpx and fish Wap65. In the present study, we confirmed the presence of *N*-linked carbohydrate chains in the Wap65-2 structure and revealed the differences in glycosylation properties between Wap65-2a and Wap65-2b. There are two glycosylation sites at positions 199 and 217 in Wap65-2a and one at position 217 for Wap65-2b; this difference affects the percentage carbohydrate moieties between Wap65-2a (29%) and Wap65-2b (27%) and their molecular masses. Similar to human Hpx, we confirmed the presence of glucosamine, sialic acid, and galactose within the carp Wap65-2 structure, but we did not detect mannose, which has been identified in human Hpx [42]. Moreover, our study indicated differences in N-terminal modification between Wap65-2a and Wap65-2b because Wap65-2a revealed N-terminal blockage that is probably caused by predicted *N*-acetylation of aspartic acid. In mammals, the N-terminal threonine residue is blocked by an *O*-linked galactosamine oligosaccharide [42]. We also observed a discrepancy between signal cleavage (amino acids 19 and 20) predicted from the obtained amino acid sequence and the obtained N-terminal sequence of Wap65-2b, which started from an aspartic acid at position 30. Similar data have been reported for catfish, where the N-terminal sequence starts from amino acid 31 instead of the predicted signal peptide cleavage between amino acids 20 and 21 [9]. It is possible that Wap65-2 contains a propeptide sequence between amino acids 20 and 29. In summary, PTMs such as glycosylation, *N*-terminal acetylation, and signal peptide/propeptide cleavage contribute to differences in molecular mass and pI between both Wap65-2a and Wap65-2b in carp seminal plasma and potentially to their structure and functions.

Our study demonstrated that both Wap65-2a and Wap65-2b comprise multiple proteoforms, each with a distinct pI. The differences in pI values (from 5.1 to 6.2) could result from PTMs that alter the protein charge, such as phosphorylation, acetylation, deamination, alkylation, oxidation, or tyrosine nitration [43]. We found 15 potential phosphorylation sites confirmed by positive reaction with Ser, Tyr, and Thr antibodies and *N*-terminal aspartic acid acetylation in the Wap65-2 structure that could result in a difference in observed pI values of Wap65-2 proteoforms. Moreover, Wap65-2 can undergo oxidative modifications such as Tyr nitration or Trp oxidation, similar to mammalian Hpx, which has been recognized as a major target of oxidation [44]. Posttranslational modifications of mammalian Hpx, such as disulfide bridge formation, glycosylation, phosphorylation, and oxidation, impact its functional properties. It should be underlined that the presence of multiple Wap65-2 proteoforms agrees with a similar phenomenon for several major fish seminal plasma proteins, such as transferrin and apolipoprotein [31, 45]. Further studies are necessary to determine the participation of PTMs in the production of multiple Wap65-2 proteoforms in carp seminal plasma and their functional importance.

Wap65-2 and Wap65-1 shared a similar tissue distribution pattern, with the highest expression in the liver; this finding is consistent with previous observations in other teleosts and mammals and suggests a pattern of liver expression that has been well conserved through evolution. In carp, Wap65-1 expression has only been identified in the liver [9]. Our study confirmed its expression in liver, although both Wap65 genes were also expressed in several other tissues/organs, including the reproductive system. To date, Wap65 expression in fish testis has been reported in pufferfish and loach [10, 18], and low Hpx expression has been detected in mammalian testis [46]. Previous studies have indicated large variations in the gene expression patterns of Wap65 isoforms in tissues among fish species, i.e., a liver-exclusive pattern versus wide distribution across diverse tissues. There have been substantial differences in the expression pattern among Wap65 isoforms in channel catfish, mud loach, pufferfish, and medaka, with Wap65-1 distributed in many tissues and Wap65-2 almost exclusively limited to the liver [8, 11, 14]. The opposite pattern has been observed for rock bream, with liver-specific Wap65-1 expression and a wide tissue distribution pattern for Wap65-2 [22]. Moreover, in turbot and loach, both Wap65 isoforms share a similar tissue distribution pattern [21]; those findings support our results.

Our study revealed Wap65-2 localization in Leydig cells, spermatogonia, and epithelium of blood vessels within the testis as well as in secretory cells of spermatic duct epithelium of carp. To the best of our knowledge, our data represent the first observation of Hpx-like protein localization in vertebrate testis. In mammalian somatic tissues, Hpx has two major functions. First, as an extracellular antioxidant, it sequesters heme and protects cells from heme-mediated oxidative stress. Second, it regulates heme delivery by receptor-mediated endocytosis to maintain cellular redox homeostasis by activation of gene expression of proteins that keep heme and iron at safe levels in cell [47]. Given that data for the reproductive system are not available, we can only speculate as to why Wap65-2 occurs in the testis. The presence of Wap65-2 in Leydig cells and early developing carp germ cells (spermatogonia) suggests that this protein participates in spermatogenesis, including maintenance of cellular redox homeostasis to promote cell survival and transport of iron to germ cells [46]. The strong reaction with the epithelium of blood vessels within the testis suggests Wap65-2 contributes to the maintenance of the blood-testis barrier and iron homeostasis; this eventuality is in agreement with Hpx functions in other physiological barriers, such as blood-brain, blood-retina, blood-follicle, and blood-ganglion barriers [4, 48]. It is possible that Wap65-2 is involved in the maintenance of blood-testis barrier through protection against heme-mediated oxidative vascular damage by scavenging heme released from hemoglobin and myoglobin as a result of hemolysis. Thus, Wap65-2 might be involved in maintenance of the blood testis-barrier integrity and delivery of iron, an action that is necessary for proper developing of germ cells and spermatozoa metabolism.

Wap65-2 localization in secretory cells of spermatic duct suggests Wap65-2 is secreted within the reproductive system, a phenomenon that can be confirmed by its presence around spermatozoa. In fish, the final spermatozoa maturation occurs in the spermatic duct, including developing the ability for movement; mature spermatozoa are maintained viable until spawning. The spermatic duct is responsible for the synthesis and secretion of enzymes, monosaccharides, lipids, and proteins into the seminal fluid [49]. Therefore, Wap65-2 localized around spermatozoa may serve as extracellular antioxidant against sperm membrane oxidation and seminal plasma proteins from damage by heme. Our earlier study indicated the presence of erythrocytes in fish seminal plasma due to injuries caused by handling fish, acquired during a spawning run, or as a result of inflammation [50]. The current results strongly suggest an important role for Wap65-2 in protecting spermatozoa from damage caused by heme released from erythrocytes following haemorrhage and inflammation.

Water temperature alters the structure and function of fish testis, including significant morphological changes [51]; however, the mechanism responsible for these changes is unknown. Our data suggest that Wap65s can be an important element of thermal adaptation in the reproductive system. Until now, the involvement of Wap65s in thermal adaptation had been determined by expression analysis restricted mainly to the liver and in some cases the head kidney, brain, gills, and muscle [10, 21, 23], but such information was not available for reproductive system. Our results indicated preferential induction of Wap65-1 expression in response to a higher temperature. The differential response to warm temperature between Wap65-1 and Wap65-2 in the testis coincides with their expression pattern in carp liver observed by Dietrich et al. [27]. This phenomenon suggests a similar mechanism of thermal acclimation within the testis and liver in carp. Our findings on the preferential induction of Wap65-1 during thermal acclimation collaborate previous observations in other fish species [19, 20, 22, 25], but the mechanism of Wap65 action during acclimation is unknown. Weber et al. [52] indicated that the warm acclimation response in fish involves increases in erythrocyte numbers and total hemoglobin content. In this context, Wap65 would be involved in heme scavenging (see above). Moreover, changes in water temperature are associated with thermal stress [53], which can trigger testicular immune response in which Wap65s have been described and discussed below.

Our results demonstrated that bacterial infection triggers the testicular immune system in carp, which was manifested by increased iNOS and pro-inflammatory cytokine IL-1β expression as well as the presence of *A. salmonicida* 16S rRNA in the testis that suggest disruption of blood-testis integrity during infection. Moreover, bacterial challenge induced a differential response between Wap65 isoforms: an increase in Wap65-2 and no change in Wap65-1. These data indicate the specificity of Wap65-2 to bacterial infection and inflammation within the testis, contrary to Wap65-1, which seems to be specific for temperature acclimation (see above). The involvement of Wap65-2 in the response to bacteria concurs with transcriptional control of Wap65-2 by factors derived from the immune system such as cytokines, e.g., IL-1 involved in induction of acute phase proteins, including Wap65 [25]. Analysis of the 5′ flanking region of carp Wap65 genes indicated the presence of more cytokine responsive elements and binding sites for transcription factors associated with immune response such as STAT, CEBP, TATABox, AP1, HNF, HSF, OCT1, IRF1, IRF2, CREBP for Wap65-2 as compared to Wap65-1 (Additional files 1, 9). The involvement of Wap65-2 in the innate immune response has been demonstrated in various tissues among fish species [14, 17, 18, 22, 26] but never in fish testis. Our results implicate Wap65-2 in testicular immune response and suggest that it can serve as antioxidant reactant during inflammation and can be involved in aiding “nutritional immunity” by sequestering heme and iron from invading pathogens to inhibit their growth.

We demonstrated an isoform-specific response of Wap65s to bacterial infection in different carp tissues. In general, bacterial infection upregulated Wap65-2 transcripts; this pattern was clear in all evaluated tissues, while the expression pattern of Wap65-1 was variable depending on tissue type, with significant downregulation in the kidney and spleen. This pattern is consistent with previous observations in channel catfish, mud loach, ayu, and rock bream: In all of those species, Wap65-2 is preferentially or exclusively induced in response to different kinds of bacterial challenges [12, 14, 17, 18, 22, 24]. Similar to our study, rock bream and flounder Wap65-1 was downregulated upon bacterial challenge, but this response depends on bacterial species and tissue types [22]. Our results indicated that depending on the tissue, Wap65 isoforms can be considered either positive or negative acute phase proteins whose expression is induced or decreased following stimulation [54]. However, the mechanism behind their significant difference in modulation pattern between two Wap65 isoforms required future studies.

In summary, two functionally distinct Wap65 isoforms are present in the carp reproductive system. Wap65-2 is a major protein of carp seminal plasma, and its presence in the reproductive system suggests a role in spermatogenesis and the protection of reproductive cells against heme-mediated oxidation during inflammation or injury. The biological role of Wap65 isoforms within reproductive system appears to be specific: Wap65-2 seems to be related to the immune response against bacteria, while Wap65-1 seems to be involved in temperature acclimation related to the teleost-lineage specificity. Our results provide important insights into the physiological role of Wap65 in the carp reproductive system and highlights their potential usefulness as biomarkers of inflammation and temperature acclimation. Moreover our work is an important first step toward the future analysis focused on determination of origin and specific function of PTMs of Wap65-2 in carp male reproductive tract.

## Supplementary information

**Additional file 1.** Methods S1–S9.

**Additional file 2:** Sequences of primers used in this work. Primers marked with ‘Q’ were used in qPCR, primers marked with ‘P’ were used for the amplification of gene fragments for plasmid based quantification of gene expression, primers marked with ‘S’ were used for sequencing of the gene.

**Additional file 3:** Identification of Wap65-2 proteoforms using MALI-TOF/TOF.

**Additional file 4:** Representative 2DE of carp seminal plasma proteome. Black frames indicate which parts of the blots were presented as Figure 1D.

**Additional file 5:** NCBI GeneBank accession numbers of sequences used for phylogenetic analysis.

**Additional file 6:** Determination of isoelectric point of Wap65-2a and Wap65-2b using isoelectrofocusing.

**Additional file 7:** Phosphoprotein detection. (A) phosphoprotein staining with Pro-Q Diamond; (B) SYPRO Ruby staining for proteins; (C) representative Western blot analysis of Wap65-2 with anti-phosphotyrosine antibodies. The 2 DE was performed on pH 4-7 strip, followed by the second dimension on 12.5% polyacrylamide gels. Std – phosphoprotein molecular weight standard; (D) representative blot analysis of Wap65-2 dephosphorylation M – molecular mass marker 202.9 -5.9 kDa; 1 – Wap65-2 after dephosphorylation with phosphatase alkaline, 2 – control preparation of Wap65-2 incubated without phosphatase alkaline.

**Additional file 8:** Isolation of Wap65-2 using hemin-agarose affinity chromatography. SP-carp seminal plasma, 1-4 unbound fractions eluted with binding buffer (10 mM sodium phosphate, pH 7.4; 0.5 M NaCl), 5–9 bound fractions containing Wap65-2 eluted with elution buffer 0.2 M sodium citrate, pH 5.2; 0.5 M NaCl and 0.02% NaN_3_).

**Additional file 9:** Transcription factor binding sites of carp Wap65-1 and Wap65-2.

## Data Availability

All data generated or analyzed during this study are included in this published article and its Additional file.

## Abbreviations

2DE: two dimensional electrophoresis
HIC: hydrophobic interaction chromatography
Hpx: hemopexin
IEC: ion exchange chromatography
MALDI TOF/TOF: matrix-assisted laser desorption/ionization time-of-flight/time-of-flight
PE: preparative electrophoresis
pI: isoelectric point
qPCR: quantitative real-time polymerase chain reaction
TBS: tris-buffered saline
Wap65: warm-temperature acclimation related 65-kDa protein
