# Supplementary material for: Characterization of carp seminal plasma Wap65-2 and its participation in the testicular immune response and temperature acclimation
Source: Vet Res. 2020 Nov 25;51:142. doi: 10.1186/s13567-020-00858-x (PMC7688007; doi:10.1186/s13567-020-00858-x)
Supplement: Supplementary file 1 — Additional file 1. Methods S1–S9. [file 13567_2020_858_MOESM1_ESM.docx]

**Method S1**

*Electrophoretic methods*

*One-dimensional gel electrophoresis (1-DE)*. After each purification step, the eluted protein fractions were analyzed by native PAGE and SDS-PAGE using an SE 250 vertical Mighty Small electrophoresis system (GE Healthcare) on a separating 12.5% acrylamide gel according to the method of Laemmli [40]. SDS-PAGE was run under nonreducing conditions, and the samples were not boiled. The gels were stained for proteins with 0.025% Coomassie brilliant blue R-250 in 40% methanol and 7% acetic acid. Molecular mass estimations were made using PageRuler Prestained Protein Ladder standards (206–7.2 kDa; Thermo Fisher Scientific, Waltham, MA, USA). The molecular weights of the proteins were estimated using the Kodak 1D program (Eastman Kodak Company).

*Two-dimensional gel electrophoresis (2-DE)*. The carp seminal plasma (30µg) or pure preparation of Wap65-2a and Wap65-2b were precipitated with the 2- DE Clean-up Kit (GE Healthcare). The pellet was resuspended in 125 μl rehydration buffer containing 7 M urea, 2 M thiourea, 2% CHAPS (3-[(3-cholamidopropyl) dimethylammonio]-1-propanesulfonatehydrate), and the protein concentration was measured. Next, 20 µg of Wap65-2a and Wap65-2b in 125 µl of 7 M urea, 2 M thiourea, 2% CHAPS, 2% pharmalyte 3 to 10 nonlinear (NL), 18 mM dithiothreitol (DTT) and a trace of bromophenol blue were loaded onto an immobiline DryStrip gel (7 cm; pH 3–10NL; GE Healthcare) with passive rehydration (15 h). Isoelectric focusing was performed at 20°C using an Ettan IPGphor apparatus (GE Healthcare), with the current limited to 50 µA/strip and the following voltage program: 300 V for 1 h, 1000 V for 0.5 h in a gradient, 5000 V for 1.5 h in a gradient, and 5000 V for 0.5 h. Before SDS-PAGE, the IPG strip was equilibrated in 6 M urea, 75 mM Tris-HCl (pH 8.8), 2% SDS, 30% glycerol, and 1% DTT (w/v) for 15 min, and then for 15 min in the same solution but with 2.5% iodoacetamide (w/v) instead of DTT and a trace of bromophenol blue. SDS-PAGE was performed using a self-cast 12.5% separating polyacrylamide gel according to the method of Laemmli [84], but without stacking gels, using an SE 250 vertical Mighty Small electrophoresis system (GE Healthcare). A run was conducted at 10 mA/gel for 0.5 h and then at 20 mA/gel until the dye reached the bottom. After electrophoresis, the gel was processed using the Coomassie staining method. To confirm that the spots corresponded to Wap65-2, each spot was separately cut off and subjected to in-gel trypsin digestion and MALDI-TOF/TOF analysis as described above.

*Protein concentration*

Protein concentrations were measured using the Bradford method and a Coomassie Plus Kit (Thermo Fisher Scientific, Waltham, MA, USA) with bovine serum albumin as a standard.

**Method S2**

*Determination of the N-terminal sequence of Wap65-2*. Samples containing 30 µg of Wap65-2 obtained after preparative electrophoresis were boiled, subjected to sodium dodecyl sulfate-polyacrylamide gel electrophoresis (SDS-PAGE), and electroblotted onto a polyvinylidene fluoride membrane using 10 mM 3-(cyclohexylamino)-1-propanesulfonic acid-NaOH (pH 11.0) containing 10% methanol. The membrane was stained with 0.1% Coomassie Brilliant Blue R-250 in 40% methanol and 1% acetic acid and was destained in 50% methanol. N-terminal protein sequence analysis was performed at BioCentrum (Kraków, Poland). The sequentially detached phenylthiohydantoin derivatives of amino acids were identified using the Procise 491 (Applied Biosystems) automatic sequence analysis system according to the manufacturer’s instructions. Sequence comparisons were performed using the SWISSProt database (<http://www.ncbi.nlm.nih.gov/blast>).

**Method S3**

*In-gel digestion and identification by MALDI TOF/TOF mass spectrometry.* Samples containing 20 µg of the pure Wap65-2a and Wap65-2b obtained after preparative electrophoresis were subjected to SDS-PAGE, and the band corresponding to 25 kDa was cut from the gel after Brilliant Blue G 250 staining and subjected to reduction, alkylation and in-gel trypsin digestion. The peptides were concentrated and desalted using ZipTip pipette tips (Sigma-Aldrich) as described previously [38]. Peptides were eluted with 1 μL of matrix solution containing 5 mg of α-cyano-4-hydroxycinnamic acid (Bruker Daltonics, Bremen, Germany) in 1 ml of 50% ACN and 0.1% TFA. This solution was spotted directly onto a steel MALDI target plate (MT 34 Target Plate Ground Steel; Bruker Daltonics). Additionally, a peptide calibration standard (Bruker Daltonics) was spotted using the dried-droplet method using the matrix for calibration of the mass spectrometer. Mass spectra were acquired in the range of 700–3500 m/z using a MALDI-TOF autoflex speed TOF/TOF mass spectrometer equipped with a Smartbeam II laser (355 nm; Bruker Daltonics). The operating conditions were the same as previously described [34, 38]. The MS spectra, together with the MS/MS spectra, were searched using the Mascot Server (Matrix Science, London, UK) of the National Centre for Biotechnology Information database. The database search criteria were as follows: enzyme: trypsin; fixed modification: carbamidomethylation (C); variable modifications: oxidation (M) peptide mass tolerance of 50 ppm, fragment mass tolerance of 0.7 Da and one missed cleavage allowed. The search results were filtered using a significant threshold of P < 0.05 and a MASCOT ion score cutoff ≥30. The same method was used for identification of protein spots from carp seminal plasma after 2DE and western blot.

**Method S4**

*Characterization of the physicochemical properties of Wap65-2*

*Determination of molecular weight*

The molecular weight of Wap65-2 was determined using a MALDI-TOF/TOF mass spectrometer (Bruker Daltonics). Before analysis, a Wap65-2 sample was desalted using ZipTip C18 (Millipore). Spectra were calibrated using the Protein Calibration Standard II (22360- 66431 Da; Bruker Daltonics*).*

*Determination of isoelectric point*

*The isoelectric point (pI) of Wap65-2 was determined after two-dimensional electrophoresis (2-DE) and isoelectrofocusing.* The isoelectric point of purified Wap65-2 (10 µg per well) was determined using isoelectric focusing (IEF). IEF was conducted under nondenaturing conditions in ready IEF Criterion™ IEF gels with a pH range of 3–10 using cathode and anode IEF buffers (Bio-Rad, ‎Hercules, CA‎, USA) for 2.5 h, starting with a constant voltage of 100 V for 1 h and then increasing to 250 V for the next 1 h and then to 500 V for 30 min. For pI calibration, the IEF standards (pI range: 4.45–9.6; Bio-Rad) were used. The isoelectric point of Wap65-2 was estimated using the Kodak 1D program.

*Enzymatic deglycosylation:* The enzymatic deglycosylation of denatured Wap65-2a and Wap65-2b was performed with N-glycosidase F (PNGase F) from Flavobacterium meningosepticum (Roche). Briefly, 6 µg of Wap65-2 was denatured by boiling in the presence of 1% SDS in 20 mM sodium phosphate buffer (pH 7.2) for 4 min. Then, 0.5% Triton X-100, 20 mM ethylenediaminetetraacetic acid (EDTA), and a proteinase inhibitor cocktail (Sigma-Aldrich Chemicals Co., St Louis, MO, USA) were added, followed by PNGase F in the proportion 2U PNGase per 10 µg protein. After 18 h of incubation at 37°C, the final reaction products were analyzed by SDS-PAGE. The Wap65-2 control samples were incubated without PNGase F. The molecular mass of deglycosylated Wap65-2 was estimated with the use of the Kodak 1D program. To check the presence of O-glycosidically linked carbohydrate chains in the structure of Wap65-2, deglycosylation with 2.5 mU O-glycosidase (Roche) was also performed as described above.

*Interaction with lectins:* The procedure described in the DIG Glycan Differentiation Kit (Roche, Penzberg, Germany) was used for the characterization of the glycoprotein moiety of Wap65-2. The interaction of Wap65-2 with the following digoxigenin-labeled lectins were tested: Galanthus nivalis agglutinin (GNA); Sambucus nigra agglutinin (SNA); Maackia amurensis agglutinin (MAA); peanut agglutinin (PNA); and Datura stramonium agglutinin (DSA). Carboxypeptidase T, transferrin, fetuin, and asialofetuin were used as control proteins (Roche). Carp Wap65-2a and Wap65-2b (1 µg) and control proteins (1 µg) were bound to a nitrocellulose membrane using the dot-blot method. Visualization of the lectins bound to Wap65-2 carbohydrate moieties was based on the staining reaction using 0.06% 5-bromo-4-chloro-3-indolyl-210 phosphate (BCIP) and 4-nitro blue tetrazolium chloride (NBT).

*Phosphoprotein detection:* Phosphoprotein detection of Wap65-2 was performed after 2-DE using phosphoprotein staining and western blot analysis. Phosphoprotein staining was performed by incubating the gel in the Pro-Q Diamond Phosphoprotein gel staining solution according to the manufacturer's protocol (Molecular Probes, Invitrogen, Eugene, Oregon, USA). Images of the stained gels were captured using a Typhoon 9400 fluorescence scanner (GE Healthcare). Then the gels were restained with SYPRO Ruby dye according to the manufacturer’s protocol (Molecular Probes) to detect proteins in the gels. The proteins separated by 2-DE were also transferred onto a nitrocellulose membrane (see Material S1). The membranes were blocked for 1 h at room temperature in TBS-T (0.05 M Tris-HCl, 0.15 M NaCl, 0.1 % Tween 20, pH 7.6) containing 1% gelatin, then incubated overnight at 4°C in 0.5% gelatin–TBS-T containing either anti-phosphoserine, anti-phosphothreonine, or anti-phosphotyrosine primary antibodies (Sigma-Aldrich).

**Method S5**

*Isolation of anti-Wap65-2 monospecific polyclonal antibodies*

The isolation procedure for anti-Wap65-2 monospecific polyclonal antibodies was performed according the method described by Wojtczak et al. 2007 which based on coupling the Wap65-2 protein as a ligand to the N-hydroxy-succinimide ester (NHS)-activated high-performance (HP) column and using this column for the affinity purification of anti-Wap65-2 monospecific IgGs.

*Coupling of Wap65-2 to the NHS-activated HP column.* The lyophilized pure Wap65-2 fraction obtained after preparative electrophoresis was suspended in the standard coupling buffer (0.2 M NaHCO_3_ and 0.5 M NaCl, pH 8.3) to a protein concentration of 1 mg/ml, and 1 ml was applied to the NHS-activated HP column (GE Healthcare). Any extra active NHS groups were deactivated by sequential washing with 0.5 M ethanolamine and 0.5 M NaCl (pH 8.3) and then with 0.1 M acetate and 0.5 M NaCl (pH 4.0). The coupled Wap65-2 was used as a ligand for the affinity purification of anti-Wap65-2 monospecific IgGs.

*Isolation procedure.* Purified total IgGs were diluted 1:1 with 100 mM Tris-HCl and 0.5 M NaCl (pH 7.6) and were applied to Wap65-2 coupled to the NHS-activated column. The unbound IgGs were washed with 50 mM Tris-HCl and 0.15 M NaCl (pH 7.6). The bound Wap65-2 monospecific IgGs were eluted with 0.5 M acetic acid (pH 3.4) and were immediately neutralized by the addition of 1 M Tris-HCl (pH 9.0). The anti-Wap65-2 monospecific IgGs were used for Western blot and immunohistochemistry analysis.

**Method S6**

*Western blot analysis using anti-Wap65-2 antibody*

Western blot analysis was performed using anti-Wap65-2 monospecific IgGs. Western blot analysis was used to check the cross-reactivity between the anti-Wap65-2 antibody and seminal plasma of carp after 1DE and 2DE and seminal plasma from different fish species barbel (*Barbus barbus* L.), dace (*Leuciscus leuciscus* L.), chub (*Squalius cephalus* L.), burbot (*Lota lota* L.), grayling (*Thymallus thymallus* L.), rainbow trout (*Oncorhynchus mykiss* Walb.), ide (*Leuciscus Idus* L.), asp *(Leuciscus Aspius* L.), Siberian sturgeon (*Acipenser baerii* Brandt, 1869) after 1DE. The samples (30 µg of protein) were applied on 12.5% SDS-PAGE gels. Western blotting was performed as described previously by Dietrich et al. [38]. Monospecific antibodies against Wap65-2 were diluted with TBS-T (0.05 M Tris-HCl, 0.15 M NaCl, 0.1% Tween 20, pH 7.4) at a ratio of 1:10000. The secondary antibody, alkaline phosphatase-goat anti-rabbit IgG conjugate (Sigma-Aldrich) was diluted 1:20000 with TBS-T, and the signal was visualized as described previously by Dietrich et al. [38].

**Method S7**

*Immunohistochemical localization of Wap65-2 in carp reproductive system and liver*

In short, to optimize immunohistochemical staining, slices were immersed in citrate buffer (10 mM, pH 6.0) and heated in a microwave oven (7 min, 650 W). Nonspecific staining was blocked twice: first with 0.3% hydrogen peroxide (H2O2) in methanol for 15 min, to inhibit endogenous peroxidase activity; and second with 5% normal goat serum for 30 min at room temperature, to block nonspecific binding sites. Thereafter, sections were incubated overnight at 4ºC in a humidified chamber in the presence of carp seminal plasma antibody against Wap65-2 (dilution 1:1,000). Subsequently, they were incubated with biotinylated secondary antibody, goat anti-rabbit IgG (1:400; Vector Lab., Burlingame CA, USA) for 60 min. After each step in these procedures, sections were carefully rinsed with Tris-buffered saline (TBS; 0.05 M Tris-HCl, 0.15 M NaCl, pH 7.6).The staining was developed using avidin biotinylated horseradish peroxidase complex (ABC/HRP; 1:100; Vectastain Elite ABC Reagent, Vector Laboratories) for 30 min. Bound antibody was visualized by 0.05% 3,3′-diaminobenzidine tetrachloride (DAB; Sigma-Aldrich) in TBS containing 0.01% H2O2 and 0.07% imidazole for 6 min. Thereafter, sections were washed and counterstained with Mayer’s hematoxylin, dehydrated, and mounted using DPX mounting media (Sigma-Aldrich). The cells were considered immunopositive if brown reaction product was present and appeared as a signal in reproductive tissue cells; the cells without any specific immunostaining were considered immunonegative [46]. All immunohistochemical experiments were repeated at least three times. Control sections included omission of the primary antibody and substitution by pre-immune goat serum. The sections were examined with a Leica DMR microscope (Leica Microsystems, GmBH Wetzlar, Wetzlar, Germany) using Nomarski interference contrast.

**Method S8**

*Hemin-agarose affinity chromatography*

Hemin agarose chromatography was performed as described by Hirayama et al. (2004). Briefly, 1 ml of hemin agarose (Sigma-Aldrich) was washed three times with binding buffer (10mM sodium phosphate, pH 7.4; 0.5 M NaCl). Carp seminal plasma was dialyzed for 24 h against binding buffer. Washed agarose was suspended in 400 µL of binding buffer and 2mL of dialyzed seminal plasma was added and incubated with agarose for 1h at room temperature. Then the mixture was centrifuged at 10 000 x g for 5 min and supernatant was removed. Hemin-agarose was washed 5 times with binding buffer to remove unbound proteins. Bound Wap65-2 was eluted after 15 min incubation with 400 µL of elution buffer (0.2M sodium citrate, pH 5.2; 0.5 M NaCl and 0.02% NaN3) and centrifugation as described above to obtain supernatant containing eluted Wap65-2. The protein fractions were analyzed using native PAGE (Fig. S4).

**Method S9**

*Transcription factor binding sites analyses*

For in silico transcription factor binding sites (TFBS) analyses of common carp wap65-2 sequence (mRNA GenBank ID: KY607421) was located on unplaced genomic scaffold of common carp genome (GenBank ID: NW_017537782) and 2108 bp region flanking start exon was selected for analyses of transcription factor binding sites. For comparison common carp wap65-1 (mRNA GenBank ID: AB052623) was located on another unplaced genomic scaffold of common carp genome (GenBank ID: NW_017540957) and 2012 bp region flanking start exon was selected for analyses of TFBS. The fragment selection was made based on wap65 TFBS analyses of rockbream (*Oplegnathus fasciatus,* Temminck & Schlegel, 1844) performed by Lee et al. 2014 [27]. TFBIND software (http://tfbind.hgc.jp/) was used for searching transcription factor binding sites using weight matrix in transcription factor database TRANSFAC R.3.4 (Wingender, 2008), with the cut-offs estimated by Tsunoda and Takagi (Tsunoda and Takagi, 1999).

Tsunoda, T., Takagi, T., 1999. Estimating transcription factor bindability on DNA. Bioinformatics 15, 622-630.

Wingender, E., 2008. The TRANSFAC project as an example of framework technology that supports the analysis of genomic regulation. Briefings in Bioinformatics 9, 326-332.
