# Supplementary material for: Characterization of carp seminal plasma Wap65-2 and its participation in the testicular immune response and temperature acclimation
Source: Vet Res. 2020 Nov 25;51:142. doi: 10.1186/s13567-020-00858-x (PMC7688007; doi:10.1186/s13567-020-00858-x)
Supplement: Supplementary file 2 — Additional file 2: Sequences of primers used in this work. Primers marked with ‘Q’ were used in qPCR, primers marked with ‘P’ were used for the amplification of gene fragments for plasmid based quantification of gene expression, primers marked with ‘S’ were used for sequencing of the gene. [file 13567_2020_858_MOESM2_ESM.docx]

**Table S1.** Sequences of primers used in this work. Primers marked with ‘Q’ were used in qPCR, primers marked with ‘P’ were used for the amplification of gene fragments for plasmid based quantification of gene expression, primers marked with ‘S’ were used for sequencing of the gene.

| **Protein name** | **Primer name** | **Primer sequence** | **Primer use** | **GenBank ID** |
| --- | --- | --- | --- | --- |
| Aeromonas spp. 16S ribosomal RNA | Aerom_16s_qF1 | GCGAAGGCGGCCCCCTGGACAAAGA | Q, P | Multiple  sequences |
|  | Aerom_16s_qR1 | CCACGTCTCAAGGACACAGCCTCCAAAT | Q, P |  |
| 40S ribosomal protein S11 | q40S.FW1 | CCGTGGGTGACATCGTTACA | Q, P | AB012087 |
|  | q40S.RV1 | TCAGGACATTGAACCTCACTGTCT | Q, P |  |
| elongation factor 1 alpha | Cyca_EF1a_qF2 | ACAACCCCAAGGCTCTCAA | Q, P | AF485331 |
|  | Cyca_EF1a_qR2 | CCGCCAACTTTCTTCTCAAC | Q, P |  |
| warm temperature acclimation-related 65 kDa protein 1 | Cyca_Wap65_1_qF | GCTGTGCAGGAATGGAGTTTG | Q, P | AB052623 |
|  | Cyca_Wap65_1_qR | CGTTTCATTAGATAGCTCTGCCTTG | Q, P |  |
| inducible nitric oxide synthase | iNOS_qF2 | CTGACTGGGTCTGGCTGGTG | Q, P | AJ242906 |
|  | iNOS_qR2 | CCTCCTCGCATTTCTCTTCTTG | Q, P |  |
| interleukin 1 beta | qIL-1β1-F | AAGGAGGCCAGTGGCTCTGT | Q, P | AJ245635 |
|  | qIL-1β1-R | CCTGAAGAAGAGGAGGCTGTCA | Q, P |  |
| warm temperature acclimation-related 65 kDa protein 2 | Cyca_Wap65_2_qF | AACTCCAGATGAAAAAGGAAACA | Q, P | KY607421 |
|  | Cyca_Wap65_2_qR | AAGGCTGCATCAACATGACC | Q, P |  |
|  | Cyca_Wap65_2_F1 | TCAGCTAAAATCAAGTGAACAGAT | S | JZ198074; EX883418 |
|  | Cyca_Wap65_2_R1 | GGGGGTTATTAAGTGTTCTTGC | S |  |
|  | Cyca_Wap65_2_F2 | TTCAGCTAAAATCAAGTGAACAG | S | JZ198074; EX883418 |
|  | Cyca_Wap65_2_R2 | TTTTAGGGATAGCCCCTTTTATG | S |  |
|  | Darecyca_Wap65_2_R3 | CTGCTGCCTTGCTCAGTCT | S | XM_005173448 |
|  | Darecyca_Wap65_2_R4 | AAACAGGATTGCTAAATCTGGA | S | XM_005173448 |
|  | Cyca_pWap65_2_F5 | CCCTGCTTCAGTGACTTTGG | S | XM_019069345 |
|  | Cyca_pWap65_2_R5 | GAAGCTCTTTCCTTGCCTTG | S |  |
