# Supplementary material for: Characterization of carp seminal plasma Wap65-2 and its participation in the testicular immune response and temperature acclimation
Source: Vet Res. 2020 Nov 25;51:142. doi: 10.1186/s13567-020-00858-x (PMC7688007; doi:10.1186/s13567-020-00858-x)
Supplement: Supplementary file 3 — Additional file 3: Identification of Wap65-2 proteoforms using MALI-TOF/TOF. [file 13567_2020_858_MOESM3_ESM.docx]

**Table S2** Identification of Wap65-2 proteoforms using MALI-TOF/TOF.

| **Spot no in Fig. 1C** | **Protein name** | **Gene ID** | **Accession**  **number** | **Calculated**  **MW/pI** | **Protein score** | **Sequence coverage**  **%** | **No of peptides** | **Precursor mass** | |  |  |
| --- | --- | --- | --- | --- | --- | --- | --- | --- | --- | --- | --- |
|  |  |  |  |  |  |  |  | **Observed** | **Theoretical** | **Peptide score** | **Peptide sequence** |
| Wap65-2a | | | | | | | | | | | |
| 1 | PREDICTED: hemopexin-like [*Cyprinus carpio*] | **HPX** | XP_018924890 | 51071/5.71 | 637 | 41 | 8 | 1870.9362  2496.1297  1185.6048  1422.6657  1438.6582  1152.5800  1816.0287  1265.6452 | 1869.9010  2495.0965  1184.5713  1421.6285  1437.6234  1151.5472  1814.9930  1264.6122 | 120  45  73  66  44  63  46  78 | K.ELDDYHLLGHVDAAFR.M  R.MHHQDDPSVHDHIYFFLDDK.V  K.GNEIYSFDIK.T  R.SYAFQDEMYIR.L  R.SYAFQDEMYIR.L Oxidation (M)  R.DGSHHFPISR.L  K.SAVHYTLIEGYPKPLK.E  K.MYDIDLAATPR.A |
| 2 | PREDICTED: hemopexin-like [*Cyprinus carpio*] | HPX | XP_018924890 | 51071/5.71 | 838 | 49 | 10 | 1870.9345  2496.1309  1185.6033  1558.7864  1422.6683  1438.6538  1152.5801  1816.0180  1265.6477  1281.6347 | 1869.9010  2495.0965  1184.5713  1557.7511  1421.6285  1437.6234  1151.5472  1814.9930  1264.6122  1280.6071 | 138  60  81  85  83  52  67  53  92  39 | K.ELDDYHLLGHVDAAFR.M  R.MHHQDDPSVHDHIYFFLDDK.V  K.GNEIYSFDIK.T  K.VWAHLPNCTSAFR.W  R.SYAFQDEMYIR.L  R.SYAFQDEMYIR.L Oxidation (M)  R.DGSHHFPISR.L  K.SAVHYTLIEGYPKPLK.E  K.MYDIDLAATPR.A  K.MYDIDLAATPR.A Oxidation (M) |
| 3 | PREDICTED: hemopexin-like [*Cyprinus carpio*] | HPX | XP_018924890 | 51071/5.71 | 172 | 27 | 1 | 1185.6070  1558.7861  1422.6682  1265.6529 | 1184.5713  1557.7511  1421.6285  1264.6122 | 34  19  21  27 | K.GNEIYSFDIK.T  K.VWAHLPNCTSAFR.W  R.SYAFQDEMYIR.L  K.MYDIDLAATPR.A |
| 4 | PREDICTED: hemopexin-like [*Cyprinus carpio*] | HPX | XP_018924890 | 51071/5.71 | 504 | 37 | 6 | 1870.9190  1185.5963  1558.7760  1422.6559  1438.6481  1152.5755  1265.6328 | 1869.9010  1184.5713  1557.7511  1421.6285  1437.6234  1151.5472  1264.6122 | 42  77  56  65  25  59  64 | K.ELDDYHLLGHVDAAFR.M  K.GNEIYSFDIK.T  K.VWAHLPNCTSAFR.W  R.SYAFQDEMYIR.L  R.SYAFQDEMYIR.L Oxidation (M)  R.DGSHHFPISR.L  K.MYDIDLAATPR.A |
| 5 | PREDICTED: hemopexin-like [*Cyprinus carpio*] | HPX | XP_018924890 | 51071/5.71 | 879 | 49 | 9 | 1870.8906  2496.0671  1185.5643  1558.7466  1134.4711  1422.6249  1438.6165  1152.5488  1815.9696  1265.6074  1281.5980 | 1869.9010  2495.0965  1184.5713  1557.7511  1133.4560  1421.6285  1437.6234  1151.5472  1814.9930  1264.6122  1280.6071 | 132  72  81  89  27  80  38  79  64  78  29 | K.ELDDYHLLGHVDAAFR.M  R.MHHQDDPSVHDHIYFFLDDK.V  K.GNEIYSFDIK.T  K.VWAHLPNCTSAFR.W  R.CEGFGHGGGEK.R  R.SYAFQDEMYIR.L  R.SYAFQDEMYIR.L Oxidation (M)  R.DGSHHFPISR.L  K.SAVHYTLIEGYPKPLK.E  K.MYDIDLAATPR.A  K.MYDIDLAATPR.A Oxidation (M) |
| 6 | PREDICTED: hemopexin-like [*Cyprinus carpio*] | HPX | XP_018924890 | 51071/5.71 | 623 | 51 | 6 | 860.4074  1870.8716  1185.5589  1438.6037  1152.5343  1281.5918 | 859.4229  1869.9010  1184.5713  1437.6234  1151.5472  1280.6071 | 56  159  66  40  68  63 | K.GNTFFFK.G  K.ELDDYHLLGHVDAAFR.M  K.GNEIYSFDIK.T  R.SYAFQDEMYIR.L Oxidation (M)  R.DGSHHFPISR.L  K.MYDIDLAATPR.A Oxidation (M) |
| Wap65-2b | | | | | | | | | | | |
| 7 | PREDICTED: hemopexin-like [*Cyprinus carpio*] | HPX | XP_018924890 | 51071/5.71 | 482 | 39 | 6 | 860.4247  1870.9136  1185.5753  1438.6370  1152.5662  1281.6167 | 859.4229  1869.9010  1184.5713  1437.6234  1151.5472  1280.6071 | 46  145  48  36  52  37 | K.GNTFFFK.G  K.ELDDYHLLGHVDAAFR.M  K.GNEIYSFDIK.T  R.SYAFQDEMYIR.L Oxidation (M)  R.DGSHHFPISR.L  K.MYDIDLAATPR.A Oxidation (M) |
| 8 | PREDICTED: hemopexin-like [*Cyprinus carpio*] | HPX | XP_018924890 | 51071/5.71 | 794 | 53 | 9 | 1870.9175  2496.1074  1185.5828  1422.6493  1438.6403  1152.5618  1815.9777  1265.6295  1281.6215 | 1869.9010  2495.0965  1184.5713  1421.6285  1437.6234  1151.5472  1814.9930  1264.6122  1280.6071 | 148  110  71  81  40  65  73  94  69 | K.ELDDYHLLGHVDAAFR.M  R.MHHQDDPSVHDHIYFFLDDK.V  K.GNEIYSFDIK.T  R.SYAFQDEMYIR.L  R.SYAFQDEMYIR.L Oxidation (M)  R.DGSHHFPISR.L  K.SAVHYTLIEGYPKPLK.E  K.MYDIDLAATPR.A  K.MYDIDLAATPR.A Oxidation (M) |
| 9 | PREDICTED: hemopexin-like [*Cyprinus carpio*] | HPX | XP_018924890 | 51071/5.71 | 718 | 42 | 9 | 860.4114  1870.9020  2496.0918  1185.5706  1422.6329  1438.6247  1152.5559  1265.6154  1281.6144 | 859.4229  1869.9010  2495.0965  1184.5713  1421.6285  1437.6234  1151.5472  1264.6122  1280.6071 | 54  153  77  81  81  36  67  76  53 | K.GNTFFFK.G  K.ELDDYHLLGHVDAAFR.M  R.MHHQDDPSVHDHIYFFLDDK.V  K.GNEIYSFDIK.T  R.SYAFQDEMYIR.L  R.SYAFQDEMYIR.L Oxidation (M)  R.DGSHHFPISR.L  K.MYDIDLAATPR.A  K.MYDIDLAATPR.A Oxidation (M) |
| 10 | PREDICTED: hemopexin-like [*Cyprinus carpio*] | HPX | XP_018924890 | 51071/5.71 | 497 | 40 | 7 | 860.4227  1870.9057  2496.0804  1185.5727  1422.6405  1438.6312  1152.5568 | 859.4229  1869.9010  2495.0965  1184.5713  1421.6285  1437.6234  1151.5472 | 34  116  43  72  64  32  58 | K.GNTFFFK.G  K.ELDDYHLLGHVDAAFR.M  R.MHHQDDPSVHDHIYFFLDDK.V  K.GNEIYSFDIK.T  R.SYAFQDEMYIR.L  R.SYAFQDEMYIR.L Oxidation (M)  R.DGSHHFPISR.L |
