# Supplementary material for: Characterization of carp seminal plasma Wap65-2 and its participation in the testicular immune response and temperature acclimation
Source: Vet Res. 2020 Nov 25;51:142. doi: 10.1186/s13567-020-00858-x (PMC7688007; doi:10.1186/s13567-020-00858-x)
Supplement: Supplementary file 4 — Additional file 4: Representative 2DE of carp seminal plasma proteome. Black frames indicate which parts of the blots were presented as Figure 1D. [file 13567_2020_858_MOESM4_ESM.docx]

**Fig. S1.** **Representative 2DE of carp seminal plasma proteome.** Black frames indicate which parts of the blots were presented as Fig. 1D.
