# Supplementary material for: Characterization of carp seminal plasma Wap65-2 and its participation in the testicular immune response and temperature acclimation
Source: Vet Res. 2020 Nov 25;51:142. doi: 10.1186/s13567-020-00858-x (PMC7688007; doi:10.1186/s13567-020-00858-x)
Supplement: Supplementary file 5 — Additional file 5: NCBI GeneBank accession numbers of sequences used for phylogenetic analysis. [file 13567_2020_858_MOESM5_ESM.docx]

**Table S3** NCBI GeneBank accession numbers of sequences used for phylogenetic analysis

| **Protein name** | **Organism** | **GenBank ID** |
| --- | --- | --- |
| Wap65-1 | *Ictalurus punctatus* | ABW07853.1 |
| Wap65-2 | *Ictalurus punctatus* | ABW07854.1 |
| Wap65-1 | *Scophthalmus_maximus* | AID59463.1 |
| Wap65-2 | *Scophthalmus maximus* | AID59464.1 |
| Wap65-1 | *Chanos chanos* | ARB08603.1 |
| Wap65-2 | *Chanos chanos* | ARB08604.1 |
| Wap65-1 | *Cyprinus carpio* | BAB60809.1 |
| Wap65-2 | *Cyprinus carpio* | ATP66527.1 |
| Wap65-1 | *Carassius auratus* | BAA08928.1 |
| hemopexin | *Carassius auratus* | XP_026060068.1 |
| Wap65-1 | *Takifugu rubripes* | BAD18109.1 |
| Wap65-2 | *Takifugu rubripes* | BAD18110.1 |
| Wap65-1 | *Oryzias latipes* | BAD98537.1 |
| Wap65-2 | *Oryzias latipes* | BAD98538.1 |
| Wap65-1 | *Lateolabrax japonicus* | CCA29189.1 |
| Wap65-2 | *Lateolabrax japonicus* | CCA29190.1 |
| hemopexin | *Danio rerio* | XP_005173505.1 |
| hemopexin | *Salmo salar* | XP_014007262.1 |
| hemopexin | *Oncorhynchus mykiss* | XP_021464322.1 |
| hemopexin | *Homo sapiens* | AAA52704.1 |
| hemopexin | *Xenopus tropicalis* | XP_002944396.2 |
| hemopexin | *Gallus gallus* | XP_015136422.1 |
| hemopexin | *Gekko japonicus* | XP_015280493.1 |
