# Supplementary material for: Characterization of carp seminal plasma Wap65-2 and its participation in the testicular immune response and temperature acclimation
Source: Vet Res. 2020 Nov 25;51:142. doi: 10.1186/s13567-020-00858-x (PMC7688007; doi:10.1186/s13567-020-00858-x)
Supplement: Supplementary file 6 — Additional file 6: Determination of isoelectric point of Wap65-2a and Wap65-2b using isoelectrofocusing. [file 13567_2020_858_MOESM6_ESM.docx]

**Fig. S2 Determination of isoelectric point** of Wap65-2a and Wap65-2b using isoelectrofocusing.
