# Supplementary material for: Characterization of carp seminal plasma Wap65-2 and its participation in the testicular immune response and temperature acclimation
Source: Vet Res. 2020 Nov 25;51:142. doi: 10.1186/s13567-020-00858-x (PMC7688007; doi:10.1186/s13567-020-00858-x)
Supplement: Supplementary file 7 — Additional file 7: Phosphoprotein detection. (A) phosphoprotein staining with Pro-Q Diamond; (B) SYPRO Ruby staining for proteins; (C) representative Western blot analysis of Wap65-2 with anti-phosphotyrosine antibodies. The 2 DE was performed on pH 4-7 strip, followed by the second dimension on 12.5% polyacrylamide gels. Std – phosphoprotein molecular weight standard; (D) representative blot analysis of Wap65-2 dephosphorylation M – molecular mass marker 202.9 -5.9 kDa; 1 – Wap65-2 after dephosphorylation with phosphatase alkaline, 2 – control preparation of Wap65-2 incubated without phosphatase alkaline. [file 13567_2020_858_MOESM7_ESM.docx]

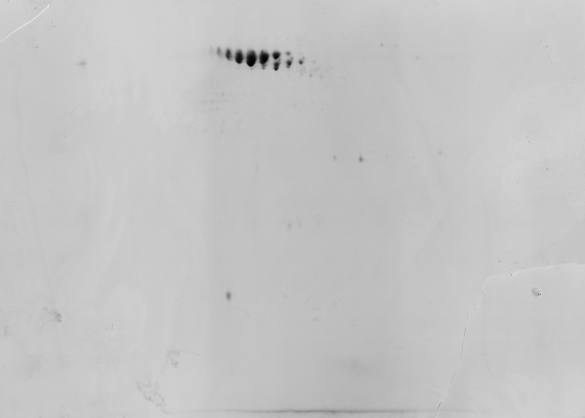

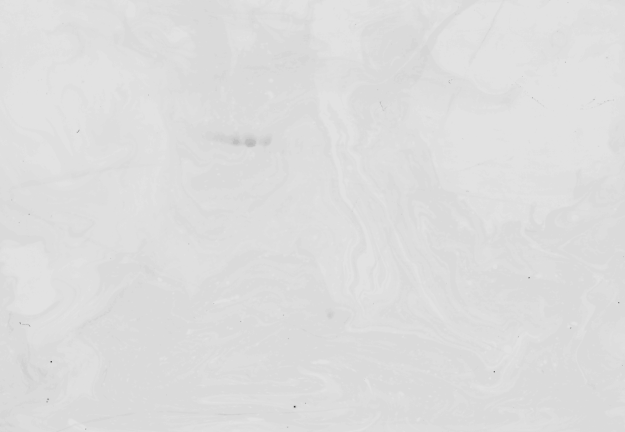


**B**

**A**


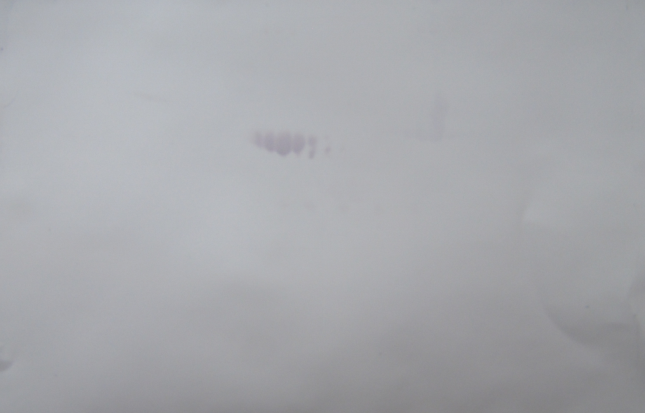


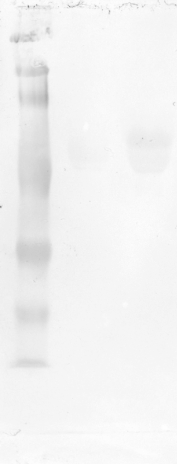


**M 1 2**

**D**

**C**

**Fig. S3.** **Phosphoprotein detection**. (A) phosphoprotein staining with Pro-Q Diamond; (B) SYPRO Ruby staining for proteins; (C) representative Western blot analysis of Wap65-2 with anti-phosphotyrosine antibodies. The 2 DE was performed on pH 4-7 strip, followed by the second dimension on 12.5% polyacrylamide gels. Std – phosphoprotein molecular weight standard; (D) representative blot analysis of Wap65-2 dephosphorylation M – molecular mass marker 202.9 -5.9 kDa; 1 – Wap65-2 after dephosphorylation with phosphatase alkaline, 2 – control preparation of Wap65-2 incubated without phosphatase alkaline.
