# Supplementary material for: Characterization of carp seminal plasma Wap65-2 and its participation in the testicular immune response and temperature acclimation
Source: Vet Res. 2020 Nov 25;51:142. doi: 10.1186/s13567-020-00858-x (PMC7688007; doi:10.1186/s13567-020-00858-x)
Supplement: Supplementary file 8 — Additional file 8: Isolation of Wap65-2 using hemin-agarose affinity chromatography. SP-carp seminal plasma, 1-4 unbound fractions eluted with binding buffer (10 mM sodium phosphate, pH 7.4; 0.5 M NaCl), 5–9 bound fractions containing Wap65-2 eluted with elution buffer 0.2 M sodium citrate, pH 5.2; 0.5 M NaCl and 0.02% NaN3). [file 13567_2020_858_MOESM8_ESM.docx]

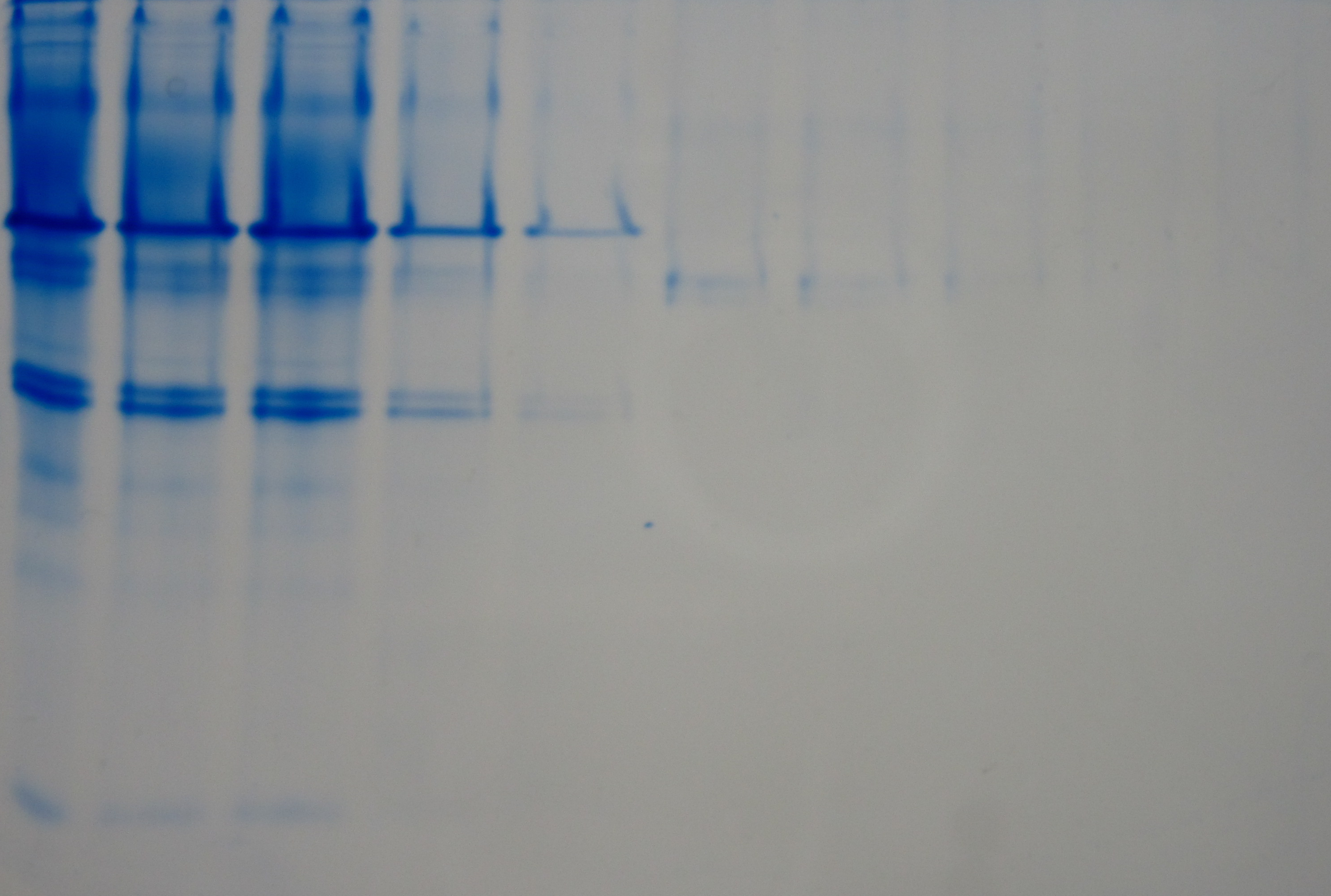


← Wap65-2

SP 1 2 3 4 5 6 7 8 9

**Fig. S4.** Isolation of Wap65-2 using hemin-agarose affinity chromatography. SP-carp seminal plasma, 1-4 unbound fractions eluted with binding buffer (10mM sodium phosphate, pH 7.4; 0.5 M NaCl), 5-9 bound fractions containing Wap65-2 eluted with elution buffer 0.2M sodium citrate, pH5.2; 0.5 M NaCl and 0.02% NaN_3_).
