# Supplementary material for: Characterization of carp seminal plasma Wap65-2 and its participation in the testicular immune response and temperature acclimation
Source: Vet Res. 2020 Nov 25;51:142. doi: 10.1186/s13567-020-00858-x (PMC7688007; doi:10.1186/s13567-020-00858-x)
Supplement: Supplementary file 9 — Additional file 9: Transcription factor binding sites of carp Wap65-1 and Wap65-2. [file 13567_2020_858_MOESM9_ESM.docx]

**Table S4.** Transcription factor binding sites of carp Wap65-1 and Wap65-2

|  | **Wap65-1** | **Wap65-2** |
| --- | --- | --- |
| NFKB | 49 | 17 |
| STAT | 13 | 22 |
| CEBP | 121 | 161 |
| TATAbox | 56 | 74 |
| AP1 | 89 | 109 |
| HNF | 45 | 74 |
| HSF | 38 | 47 |
| OCT1 | 206 | 217 |
| SP1 | 67 | 20 |
| IRF1 | 11 | 15 |
| IRF2 | 12 | 17 |
| USF | 69 | 47 |
| CREBP | 52 | 67 |
